# The role of the gut mycobiota in neurodevelopmental disorders: a multikingdom disruption of the gut-brain axis

**DOI:** 10.3389/fmicb.2026.1798439

**Published:** 2026-05-07

**Authors:** Yue Hu, Xiao-Li Yu, Xia-Jing Zhang, Li-Tian Ma

**Affiliations:** 1 College of Teacher Education, Shaanxi University of Technology, Hanzhong, Shaanxi, China; 2 Clinical Psychology Program, Department of Psychology, School of Graduate Studies, Integrated Research Track in Psychopathology and Neuropsychology, Bio–Psycho–Medical Interdisciplinary Research Group, Independent Chinese Personal Research Unit, Manuel L. Quezon University, Quezon City, Philippines; 3 Department of Internal Medicine, Tangdu Hospital, Fourth Military Medical University (Air Force Medical University), Xi’an, China; 4 Department of Anesthesiology, The Second Affiliated Hospital of Shaanxi University of Chinese Medicine, Xianyang, Shaanxi, China; 5 Department of Gastroenterology, Tangdu Hospital, Fourth Military Medical University (Air Force Medical University), Xi’an, China; 6 Department of Thoracic Surgery, Tangdu Hospital, Air Force Medical University, Xi’an, China; 7 School of Medicine, Northwest University, Xi’an, China

**Keywords:** fungal dysbiosis, intestinal barrier dysfunction, neuroinflammatory pathways, short-chain fatty acids, systemic immune activation

## Abstract

Research on the gut-brain axis in neurodevelopmental disorders (NDDs) has historically focused on bacteria, overlooking fungal contributions. This review synthesizes emerging evidence to propose that intestinal fungi may actively contribute to NDD pathophysiology through multikingdom interactions, though causality requires validation. We delineate a consistent gut mycobiota dysbiosis signature across autism spectrum disorder (ASD), attention-deficit/hyperactivity disorder (ADHD), and Rett syndrome (RTT) characterized by reduced diversity and Candida expansion. Potential mechanisms include compromised intestinal barrier integrity, systemic immune activation via Dectin-1/Syk/CARD9 signaling, and disruption of neuroactive metabolites like short-chain fatty acids. We propose distinct etiological pathways: a “top-down” cascade in RTT, where *MeCP2* mutation-induced dysmotility creates a pro-dysbiotic niche, and a “bottom-up” pathway in ASD and ADHD, where bacterial dysbiosis erodes colonization resistance, permitting fungal overgrowth. This “bacteria–fungi–host” framework provides a coherent explanation for gut-brain axis disruptions and opens avenues for biomarker discovery and multikingdom therapeutic interventions.

## 1 Introduction

Core neurodevelopmental disorders (NDDs), including autism spectrum disorder (ASD), attention-deficit/hyperactivity disorder (ADHD), and Rett syndrome (RTT), are characterized by early brain maturation disruptions (Sokolowski and Levine, 2022; Strati et al., 2016; Wang Q. et al., 2023). Historically, NDD research focused on genetic models and neuronal circuit defects (Khodosevich and Sellgren, 2023). However, incomplete heritability and environmental factors have shifted contemporary frameworks toward gene–environment interactions.

The discovery of the bidirectional communication network between the gut and the brain—the gut-brain axis—has fundamentally transformed our understanding of brain development and function (Wang Q. et al., 2023). The gut microbiota can modulate key neurodevelopmental and homeostatic processes, including neuroimmune signaling, myelination, and the refinement of neural circuits through mechanisms such as synaptic pruning (Wang Q. et al., 2023). Research in this field has predominantly focused on bacterial communities, elucidating how certain species (e.g., specific strains of *Bifidobacterium* and *Lactobacillus*) produce metabolites like short-chain fatty acids (SCFAs) and engage neural pathways such as the vagus nerve to influence host physiology and behavior (Wang Q. et al., 2023). This predominantly bacteriocentric paradigm, however, presents a conceptual limitation. This regulation extends beyond immunomodulation and barrier maintenance. A pivotal, well-established mechanism is the direct microbial production of neuroactive metabolites. Gut bacteria synthesize a range of compounds—including the primary inhibitory neurotransmitter gamma-aminobutyric acid (GABA), the essential cofactor biotin, and precursors to pivotal neuromodulators like dopamine—that can influence neuronal excitability, synaptic plasticity, and behavior (Strandwitz, 2018; Wang Q. et al., 2023). This metabolic output constitutes a fundamental chemical dialogue within the microbiota-gut-brain (MGB) axis. However, the prevailing bacteriocentric lens has predominantly framed these metabolites as products of bacterial metabolism, overlooking the ecological context in which they are produced. A holistic understanding necessitates a paradigm shift to a multikingdom perspective. Critically, the stability and functional output of the bacterial communities that produce these neuroactive compounds are themselves regulated by intricate cross-kingdom interactions. Within this framework, the gut mycobiota emerges as a potent modulator. Fungi, despite their low relative abundance, can function as keystone species, disproportionately influencing the structure and function of the entire gut ecosystem through direct competition, alteration of the local microenvironment, and modulation of host immunity (Gaspar et al., 2025; Li et al., 2019). Consequently, fungal dysbiosis may disrupt the bacterial synthesis of key neuroactive metabolites, not merely as a bystander but as an active upstream disruptor. Therefore, a comprehensive “bacteria-fungi-host” interaction model is essential to unravel how disruptions in this tripartite dialogue contribute to neurodevelopmental pathology. The gut is a multikingdom ecosystem in which bacteria, fungi, viruses, and archaea engage in continuous cross-kingdom interactions (Gilbert et al., 2018). Fungi, in particular, can function as keystone species—disproportionately influential regulators of community structure and function despite their low relative abundance (Gutierrez and Arrieta, 2021; Mohammadi et al., 2025). Consequently, a holistic understanding of the MGB axis necessitates integrating the mycobiota, as fungal perturbations can exert profound direct effects on host immunity and barrier function, while also critically modulating bacterial ecology to precipitate or exacerbate dysbiosis (Gutierrez and Arrieta, 2021; Mohammadi et al., 2025).

Fungal DNA constitutes ∼0.1%–1% of total gut microbial DNA (Shuai et al., 2022), historically characterizing gut mycobiota as “dark matter.” However, low abundance does not equate to minor significance. Fungi exert disproportionate functional impacts on host immunity and metabolism (Sam et al., 2017; Shuai et al., 2022) *via* two key mechanisms: (1) fungal cell walls contain unique pathogen-associated molecular patterns (PAMPs) (β-glucans, chitin) recognized by specific pattern recognition receptors (PRRs) (Dectin-1, Toll-like receptors (TLRs)), initiating inflammatory cascades (IL-17, IL-22) (McBride et al., 2023; Patin et al., 2019; Sey et al., 2024); (2) morphological plasticity allows Candida to switch from yeast to invasive hyphal forms, disrupting epithelial barrier integrity and facilitating microbial translocation (Patin et al., 2019).

This transition not only enhances tissue adhesion and invasion but can also directly disrupt the integrity of the intestinal epithelial barrier, facilitating microbial translocation and systemic immune activation (Patin et al., 2019). Consequently, in the context of NDDs, fungal dysbiosis may represent more than a quantitative imbalance in the community composition, although whether it serves as a primary causative factor or a secondary consequence remains to be determined through prospective studies (Hadrich et al., 2024; Loh et al., 2024). Within this framework, fungal dysbiosis in NDDs has been conceptualized as a state of functional maladaptation of the gut ecosystem (Hadrich et al., 2024; Loh et al., 2024). This state is characterized by three interrelated shifts: (1) a loss of fungal α-diversity, often involving a depletion of putative beneficial commensals (e.g., *Saccharomyces* spp.) (Loh et al., 2024); (2) an expansion of opportunistic pathobionts (e.g., *Candida albicans*) (Loh et al., 2024); and (3) an alteration in the metabolic output of the community, including increased production of metabolites (e.g., ammonia and certain D-arabinose isomers) that can disrupt host neuroimmune and metabolic homeostasis (Hadrich et al., 2024; Loh et al., 2024).

At a conceptual level, the fungal components discussed above (e.g., β-glucans and chitin) are traditionally termed PAMPs in host–fungal interactions, highlighting their role in triggering anti-pathogen defenses. However, within the complex ecosystem of the gut microbiota, comprising commensals, pathobionts, and fungi, the more inclusive term microbial-associated molecular patterns (MAMPs) is increasingly adopted. This conceptual shift reflects the consensus that the same molecules can function as either harmless stimuli or proinflammatory signals, depending on the microbial context, barrier integrity, and host immune status. In the specific setting of fungal dysbiosis addressed here, where commensal-like fungi can exert a proinflammatory effect without being classic pathogens, the term MAMPs more accurately describes the ligands involved in disrupting immune homeostasis.

Despite its potential importance, progress in characterizing the human mycobiome has been hampered by methodological challenges that critically impact reproducibility and cross-study comparisons (Sam et al., 2017; Strati et al., 2017). The central issue is the contrast between sequencing targets: while bacterial analysis relies on the standardized 16S rRNA gene, fungal studies use the highly variable internal transcribed spacer (ITS) region. This variability, along with severe primer-specific amplification bias, can systematically skew taxonomic results, complicating direct comparisons between studies that use different methods (Sam et al., 2017; Strati et al., 2017). These challenges are compounded by less comprehensive fungal databases, which often restrict analysis to the genus level, and the low absolute biomass of fungi, which increases susceptibility to contamination (Sam et al., 2017; Strati et al., 2017). Therefore, it is essential to interpret the data synthesized in this review with caution, acknowledging that methodological heterogeneity may contribute to apparent discrepancies. However, recent technological advances are beginning to overcome these hurdles, facilitating a more precise characterization of the gut mycobiome. This review synthesizes these emerging data to critically appraise the associative evidence linking fungal community dynamics to NDDs. While the discussion explores specific mechanistic pathways, most prominently the immune cascade model, through which fungal dysbiosis is hypothesized to potentially act as an active contributor rather than a passive correlate in neurodevelopmental pathology, we emphasize that these proposed mechanisms are based primarily on associative and preclinical evidence. Definitive causal relationships await validation through longitudinal human studies and controlled interventional trials.

## 2 Linking gut bacterial–fungal dysbiosis to systemic consequences

The evidence from experimental studies suggests that the human gut can be characterized as a complex, multikingdom ecosystem, where the commensal status of the mycobiota is often modulated by dynamic interactions with the bacteriome, particularly under homeostatic conditions (Huseyin et al., 2017; Iliev et al., 2012). In NDDs such as ASD, the evidence suggests that the destabilization of this interface—often characterized by bacterial dysbiosis—may facilitate fungal expansion and subsequent mucosal or systemic inflammation (Strati et al., 2017).

### 2.1 Colonization resistance as an ecological constraint on fungi

In a homeostatic intestinal ecosystem, fungal colonization (e.g., by *Candida* species) is constrained by a mechanism known as colonization resistance, which is primarily established by commensal bacteria (Deveau et al., 2018). This resistance limits the ecological niche of fungi through direct competition and immune regulation (Deveau et al., 2018). For instance, the recognition pathways mediated by host immune receptors such as Dectin-1 are an important component of immune regulation under the influence of bacterial communities (Iliev et al., 2012).

Current research, particularly from metagenomic analyses, is beginning to elucidate specific bacteria-mediated mechanisms that are thought to help maintain fungi in a commensal state. One proposed immune-mediated containment pathway is the aryl hydrocarbon receptor (AhR)–interleukin-22 (IL-22) axis (Kang et al., 2025). This model suggests that commensal bacteria, such as *Lactobacillus* species, can contribute to host immune responses by metabolizing dietary tryptophan into AhR ligands (Huang et al., 2023). The binding of these ligands to the AhR on Group 3 innate lymphoid cells (ILC3s) is hypothesized to stimulate the secretion of IL-22 (Huang et al., 2023; Kang et al., 2025). Furthermore, fungal antigens may promote activation of IL-17-producing T-helper 17 (Th17) cells, enabling cooperation with the AhR–IL-22 pathway to restrict fungal proliferation (Sparber and LeibundGut-Landmann, 2019). This coordinated response is hypothesized to help prevent fungal epithelial invasion and maintain homeostasis.

Commensal bacteria constrain fungal proliferation through bioactive metabolites that alter the intestinal microenvironment. SCFAs (acetate, propionate, butyrate) regulate gut mycobiota through four mechanisms: (1) acidifying the colonic lumen (pH 5–6), inhibiting pH-sensitive fungi (Ríos-Covián et al., 2016); (2) butyrate fuels colonocytes, reinforcing epithelial barrier via mucin production and tight junction upregulation (STAT3/SP1 pathways) (Parada Venegas et al., 2019); (3) suppressing yeast-to-hyphal transition (Gaspar et al., 2025; McCrory et al., 2023); (4) modulating mucosal immunity via histone deacetylases (HDACs) inhibition and Treg differentiation (Gaspar et al., 2025; Loh et al., 2024; McCrory et al., 2023; Parada Venegas et al., 2019). Depletion of SCFA-producing taxa in dysbiosis compromises these constraints, creating a permissive niche for fungal expansion (Cheung et al., 2019; Parada Venegas et al., 2019).

Ultimately, this intricate network of metabolic and immune-mediated checkpoints renders the mycobiota highly sensitive to bacterial fluctuations. Any disruption to the bacteriome—driven by antibiotics or environmental stressors—can compromise these dual layers of colonization resistance, effectively destabilizing the ecosystem and creating a permissive niche for fungal pathogenesis (Hadrich et al., 2024; Strati et al., 2017).

### 2.2 Fungal expansion driven by niche release and dysbiosis

The collapse of bacterial colonization resistance provides an ecological opening for fungal dysbiosis. In NDDs, this is conceptualized as “niche release”—where depletion of competitive bacterial taxa eliminates inhibitory pressures, allowing opportunistic fungi to proliferate with reduced inhibitory constraints (Gaspar et al., 2025; Severance et al., 2016).

Autism spectrum disorder clinical studies reveal that gut bacterial alterations (elevated *Firmicutes*/*Bacteroidetes* ratio, reduced *Bacteroidetes*) associate with mycobiota changes. Although fungal expansion (e.g., 2-fold *Candida* increase) did not reach high significance in Strati et al. (2017), findings support a model where bacterial dysbiosis fosters a permissive environment for fungal alterations (Strati et al., 2017). Depletion of beneficial taxa (*Alistipes*, *Bilophila*, *Dialister*, *Veillonella*) alongside increases in *Collinsella* and *Corynebacterium* have been noted (Gaspar et al., 2025; Strati et al., 2017) Reduced *Bacteroidetes* and *Lactobacillus* result in scarcity of tryptophan-derived ligands and SCFAs necessary to maintain immune/metabolic checkpoints (IL-22 axis, pH regulation) that suppress fungal overgrowth (Hadrich et al., 2024; Huang et al., 2023; Kang et al., 2025; Severance et al., 2016).

Consequently, bacterial dysbiosis can trigger profound shifts in the mycobiota through disrupted interkingdom interactions, a dynamic that is increasingly recognized in neuropsychiatric disorders. This inter-kingdom relationship is exemplified in ASD. Strati et al. (2017) provided seminal evidence of an altered fungal community composition in patients with ASD, characterized by a predominance of families like *Saccharomycetaceae* and *Debaryomycetaceae* (Strati et al., 2017). While the role of *Candida* species in ASD may be more limited compared to other taxa, its expansion, where observed, is significant. Critically, such mycobiota alterations may be associated with the pathology of the disorder and could potentially influence neurobehavioral symptoms via gut-brain axis mechanisms, including effects on gut permeability, immune responses, and microbial metabolite production. However, whether these alterations represent a primary causative factor or a secondary consequence of gastrointestinal symptoms like constipation—or a combination of both—remains to be established through longitudinal studies (Hadrich et al., 2024; Strati et al., 2017). Furthermore, the pronounced bacterial–fungal dysbiosis in patients with RTT, which is characterized by co-occurring proinflammatory bacteria and *Candida* overgrowth, establishes a dysbiotic ecosystem linked to local inflammation and altered neuroactive metabolite profiles, underscoring the broad relevance of interkingdom dynamics across neurodevelopmental disorders (Strati et al., 2016). Further complexity was added by Zou et al. (2021), who identified that while *Candida* expands, other fungal populations also undergo significant restructuring; specifically, they observed a significant increase in the abundance of Saccharomyces cerevisiae (58.38% in patients with ASD vs. 36.72% in control subjects) (Zou et al., 2021). These findings suggest that the mycobiota in patients with ASD does not merely experience a uniform overgrowth but rather a selective expansion of specific taxa capable of exploiting the dysbiotic bacterial niche.

Similar patterns of dysbiosis are observed in patients with other NDDs, such as RTT, where the host genetic background, particularly MeCP2 mutations, is associated with distinct alterations in the gut microbiota and mycobiota. In RTT patients, the fungal community is characterized by a significant increase in the relative abundance of *Candida* (61.3% in patients with Rett syndrome vs. 25.5% in healthy controls) alongside reduced microbial diversity, which occurs in the context of a systemic sub-inflammatory state marked by elevated fecal calprotectin levels and erythrocyte sedimentation rates (Strati et al., 2016). These microbial shifts are functionally consequential: the reduced levels of beneficial bacteria and their metabolites, such as SCFAs, may diminish competitive inhibition and immunoregulatory mechanisms that normally constrain fungal growth. In such a permissive environment, opportunistic fungi like *Candida* can transition from commensalism to increased abundance and potential pathogenicity. This transition is facilitated not only by the loss of bacterial suppression but also by alterations in the intestinal milieu, such as elevated levels of propionate and branched-chain fatty acids, which have been linked to impaired gut motility and mucosal function in patients with RTT (Strati et al., 2016). The resulting fungal expansion may enhance mucosal adhesion and invasion, thereby exacerbating gastrointestinal symptoms and contributing to persistent immune activation, as evidenced by the correlation between fungal dysbiosis and inflammatory markers in clinical studies (Gaspar et al., 2025; Hadrich et al., 2024; Strati et al., 2016).

Building upon the ecological framework of “niche release” described above, where the erosion of bacterial colonization resistance permits opportunistic fungal expansion, examining how these microbial shifts manifest within specific clinical populations is crucial. Emerging evidence indicates that fungal dysbiosis is not merely a passive by-product of bacterial disruption but an active contributor to the aetiology and progression of NDDs (Gaspar et al., 2025). In this section, we explore the fungal alterations observed in patients with ASD, ADHD, and RTT. Despite the heterogeneity of these conditions, a recurring pattern of fungal dysbiosis that is often characterized by *Candida* overrepresentation suggests common mechanistic pathways (Forbes et al., 2019; Hadrich et al., 2024; Hughes and Ashwood, 2018; Strati et al., 2017). We specifically analyse how these fungal imbalances drive pathology through compromised gut barrier integrity, chronic systemic inflammation, and the dysregulation of neuroactive metabolites, thereby influencing specific phenotypes associated with these disorders.

## 3 Evidence and mechanisms of gut mycobiota dysbiosis in patients with ASD

### 3.1 Evidence of an altered fungal composition in patients with ASD

Recent studies on intestinal mycobiota in ASD patients reveal complex alterations contributing to disease mechanisms (Hadrich et al., 2024; Kantarcioglu et al., 2016; Strati et al., 2017). Metataxonomic analyses demonstrate structural changes in intestinal fungal populations. Strati et al. (2017) showed *Candida* genus abundance more than doubled in autistic subjects versus neurotypical controls (37.7% vs. 14.1%), though high dispersion yielded only partial significance (Welch *t*-test, FDR-corrected *P* = 0.09). Beta diversity analysis revealed significant group separation (*P* < 0.05, PERMANOVA) (Strati et al., 2017), with *Saccharomycetaceae* and *Debaryomycetaceae* families showing altered abundance potentially linked to gastrointestinal symptoms (Strati et al., 2017). Specific fungal families including *Saccharomycetaceae* and *Debaryomycetaceae* showed altered abundance patterns in ASD cohorts, potentially linking these mycobiota changes to the gastrointestinal symptoms frequently observed in this population (Strati et al., 2017). Longitudinal evidence from the study by Kantarcioglu et al. (2016) strengthens these observations, with laboratory data spanning 17 years showing a high prevalence of yeast isolated from pediatric ASD patients (72.7% of 1,555 samples). Among these isolates, *C. albicans* predominated (57.4%), and a subset analysis confirmed significantly higher yeast detection rates in ASD patients (81.4%) than in healthy controls (19.6%, *P* < 0.05) (Kantarcioglu et al., 2016). Notably, species with intrinsic azole resistance—*C. krusei* (19.8%) and *C. glabrata* (14.8%)—were identified exclusively in samples from patients with ASD, raising important considerations for potential therapeutic approaches (Kantarcioglu et al., 2016).

The relationship between fungal presence and clinical manifestations appears nuanced. While some studies correlate *C. albicans* with exacerbation of gastrointestinal disturbances in patients with ASD (Strati et al., 2017), emerging evidence suggests that fungal dysbiosis is not confined to ASD subgroups with gastrointestinal symptoms. A pilot study by Alookaran et al. (2022) found no significant differences in *Candida* prevalence between children with ASD and gastrointestinal symptoms (21%), those without gastrointestinal symptoms (56%), and typically developing controls (25%) (overall *P* = 0.18), and *Candida* abundance did not correlate with behavioral or gastrointestinal symptom severity (Alookaran et al., 2022). Similarly, Hughes and Ashwood (2018), Vautier et al. (2012) reported that elevated anti-*C. albicans* IgG antibodies in children with ASD were not strictly correlated with current gastrointestinal symptoms, implying that fungal-driven immune activation could occur even in the absence of overt gastrointestinal distress. Furthermore, Zeng et al. (2025) demonstrated that the gut mycobiota composition differed between children with severe and mild ASD, with *Candida* being significantly elevated only in the severe ASD subgroup (67.52% vs. 4.45% in neurotypical controls), suggesting that fungal dysbiosis may be more closely associated with ASD severity than with gastrointestinal comorbidities *per se*. Collectively, these findings characterize ASD-related gut mycobiota dysbiosis as featuring (1) a distinct community structure, (2) increased prevalence of specific yeast species including non-albicans Candida with potential clinical relevance, and (3) alterations in commensal fungal populations, all of which potentially contribute to the complex pathophysiology of ASD through the gut-brain axis.

It is important to acknowledge that the fungal landscape in ASD is more nuanced than a simple pathogenic enrichment model. While *Candida* species are frequently elevated in ASD cohorts, emerging evidence indicates that *Candida albicans* could maintain commensal relationships with the host and contribute to gut homeostasis under specific conditions (Peroumal et al., 2022). Indeed, large-scale metagenomic analyses have revealed that the majority of fungal species (80 out of 90 identified) are actually depleted rather than enriched in children with ASD (Su et al., 2024). Furthermore, *Saccharomyces* abundance is typically higher in neurotypical individuals and reduced in severe ASD (Su et al., 2024), while *Aspergillus* species show distinct abundance patterns in ASD compared to controls (De Santis et al., 2019; Su et al., 2024). These findings underscore that fungal dysbiosis in ASD may reflect not merely the overgrowth of pathogenic species but also the loss of commensal fungal populations that support gut integrity (Wu et al., 2025).

### 3.2 Mechanistic insights underlying fungal dysbiosis in patients with ASD

The potential mechanisms through which altered fungal communities contribute to autism spectrum disorder are multifaceted. Research indicates that the intestinal mycobiome can interact with the host’s immune system, metabolic processes, and even gene expression regulation. Here, we outline several key mechanistic pathways based on the current literature.

#### 3.2.1 Intestinal permeability and barrier function: heterogeneity and fungal involvement

Intestinal epithelial barrier integrity remains debated in ASD pathophysiology. While the “leaky gut” hypothesis—uncontrolled translocation of microbial products into circulation—is frequently cited, clinical evidence is heterogeneous. Early studies reported abnormal intestinal permeability in ∼37% of children with ASD versus <5% of controls, rising above 40% in those with gastrointestinal symptoms (de Magistris et al., 2010; Fattorusso et al., 2019).

However, subsequent well-controlled investigations have challenged the universality of this dysfunction. For instance, Dalton et al. (2014) utilized the lactulose/mannitol (L/M) recovery ratio—a gold standard for permeability assessments—and found no significant differences between children with ASD and age-matched controls with special educational needs (mean L/M ratios: 0.015 vs. 0.014, respectively). Similarly, Kushak et al. (2016) reported no statistically significant difference in permeability between children with ASD and non-autistic children when controlling for gastrointestinal symptoms, suggesting that barrier dysfunction may be more closely associated with general gastrointestinal distress rather than ASD *per se*. Notably, Alookaran et al. (2022) found no significant elevation in fecal calprotectin or serum Dectin-1 levels in children with ASD compared to controls, further challenging the notion that fungal dysbiosis is invariably linked to gut inflammation in ASD. These discrepancies indicate that increased intestinal permeability is likely not a ubiquitous feature of ASD but rather a pathological trait specific to a subset of individuals, particularly those with severe ASD phenotypes or specific inflammatory profiles, rather than being exclusively associated with gastrointestinal comorbidities.

In the subset of individuals where barrier integrity is compromised, fungal dysbiosis is posited to act not merely as a passive observer but as an potential active modulator of epithelial function. *C. albicans*, the most prevalent fungal pathobiont, possesses distinct mechanistic capabilities to exploit and exacerbate barrier vulnerability. (1) Direct mechanical and cytotoxic disruption: through morphological plasticity, *C. albicans* transitions from a commensal yeast to an invasive hyphal form. This transition, as demonstrated by Dalle et al. (2010), facilitates physical adhesion to the epithelium and penetration of epithelial cell layers *via* both induced endocytosis and active penetration, ultimately leading to compromised epithelial barrier function. Concurrently, fungal metabolic activity produces cytotoxic compounds, such as ammonia, which can damage the mucosal lining and further weaken epithelial coherence (Ene et al., 2014). (2) Immunological modulation of tight junctions: the morphological plasticity of *C. albicans* enables its transition from a commensal yeast to an invasive hyphal form. This transition is associated with increased adhesion to epithelial surfaces and tissue penetration. Crucially, the hyphal form exposes specific PAMPs, which are recognized by host pattern recognition receptors (PRRs) on epithelial and immune cells. This recognition triggers potent proinflammatory signaling cascades (e.g., *via* NF-κB), leading to the production of cytokines such as IL-1β and TNF-α. These inflammatory mediators are known to downregulate the expression and alter the organization of tight junction proteins, thereby compromising epithelial barrier integrity. Thus, the hyphal transition contributes to barrier dysfunction primarily by initiating a host inflammatory response that disrupts tight junction complexes, rather than through direct physical disruption alone.

Consequently, while fungal dysbiosis may not be the primary initiator of barrier dysfunction in all patients with ASD, it likely functions as a critical modifier in vulnerable individuals. A bidirectional feedback loop may emerge in this specific subgroup: an initial genetic or environmentally induced barrier defect creates a niche permissive for fungal overgrowth; established fungal populations then employ direct invasion and inflammatory signaling to further degrade the barrier. This cycle facilitates the translocation of microbial metabolites and neuroactive compounds, potentially linking localized gut dysfunction to the systemic immune activation and neurobehavioral exacerbations observed in patients with the ASD phenotype.

#### 3.2.2 Immune dysregulation and systemic inflammation

Beyond the gut mucosa, fungal dysbiosis in ASD is implicated in systemic immune dysregulation. The immune response to fungal overgrowth, particularly *Candida* species, creates a chronic inflammatory milieu paralleling immune profiles observed in ASD subsets mediated by fungal antigen-host immune interactions driving proinflammatory signaling (Hughes and Ashwood, 2018).

The primary mechanism linking fungal dysbiosis to systemic inflammation involves the recognition of fungal cell wall components, particularly β-glucans, by PRRs on host immune cells. Dectin-1, a C-type lectin receptor expressed on dendritic cells and macrophages, binds β-glucans and activates the spleen tyrosine kinase–caspase recruitment domain-containing protein 9 (Syk–CARD9) signaling axis (Brown, 2006; Iliev et al., 2012). This pathway leads to assembly of the Bcl10–MALT1 complex, ultimately activating NF-κB to induce expression of proinflammatory cytokines including IL-6, IL-23, IL-1β, and TNF-α (Iliev et al., 2012; Li et al., 2019). This signaling cascade acts synergistically with TLR stimulation to activate the NACHT, LRR and PYD domains-containing protein 3 (NLRP3), which processes pro-cytokines into their mature, active forms (Brown, 2006; Gringhuis et al., 2012; Iliev et al., 2012; Li et al., 2019). The critical role of CARD9 in antifungal immunity is underscored by human CARD9 deficiency—the only known inborn error of immunity that predisposes to both mucosal and invasive candidiasis with unique CNS tropism (Lionakis et al., 2023).

Notably, the immune response to fungal pathogens is intrinsically linked to the Th17 pathway. *Candida* antigens stimulate the differentiation of naïve T cells into Th17 cells, which produce interleukin-17 (IL-17) and IL-22 (Acosta-Rodriguez et al., 2007). While IL-17 is essential for acute mucosal defences against fungal infections, its chronic elevation is pathogenic (Wu et al., 2021). Sustained high levels of IL-17 suppress the function of regulatory T cells (Tregs), thereby eroding immune tolerance and promoting continuous tissue damage and autoimmunity (Wang Q. et al., 2025; Zheng et al., 2020). Elevated IL-17 levels are a reproducible finding in patients with ASD (Gładysz et al., 2018) and have been mechanistically linked to neurodevelopmental deficits in maternal immune activation (MIA) models (Choi et al., 2016; Gładysz et al., 2018). The evidence from animal models suggests a potential link between interleukin-17A (IL-17A) and neurodevelopment: in MIA studies, circulating IL-17A may cross the placental barrier during gestation and possibly the BBB, binding to interleukin-17 receptor A (IL-17RA) expressed on neural cells, including neurons and microglia (Choi et al., 2016; Gładysz et al., 2018). This binding is associated with microglial activation and neuroinflammatory responses, which could contribute to synaptic dysfunction (Choi et al., 2016; Gładysz et al., 2018). In experimental settings, these processes have been linked to cortical disorganization and altered neural connectivity, correlating with behavioral abnormalities like social deficits and repetitive behaviors observed in individuals with ASD (Choi et al., 2016; Gładysz et al., 2018). Immune dysregulation, as highlighted in clinical studies, may perpetuate IL-17-mediated neuroinflammation, although direct human evidence for barrier penetration remains limited (Choi et al., 2016; Gładysz et al., 2018). Consequently, fungal dysbiosis may serve as a continuous endogenous source of IL-17, potentially contributing to a neuroinflammatory state, though direct causal evidence in human subjects is currently limited.

Furthermore, the assertion that intestinal fungal dysbiosis leads to systemic immune activation is supported by serological evidence. A study by Hughes and Ashwood (2018) identified significantly elevated levels of anti-*C. albicans* IgG antibodies in the plasma of children with ASD compared to neurotypical controls (36.5% vs. 14.3% positivity). The presence of IgG antibodies indicates a systemic adaptive immune response to fungal antigens, suggesting that these antigens have likely breached the intestinal barrier to interact with circulating immune cells (Hughes and Ashwood, 2018). Importantly, this antibody response was not strictly correlated with current gastrointestinal symptoms, implying that fungal-driven immune activation can occur even in the absence of overt gastrointestinal distress, acting as a “silent” driver of systemic dysregulation (Hughes and Ashwood, 2018).

Additionally, the chronic antigenic load from fungal dysbiosis further disrupts the homeostatic balance between proinflammatory cells and Tregs. The persistent drive toward Th1 and Th17 differentiation in response to fungal overgrowth comes at the expense of Treg populations (Bacher et al., 2014; Li et al., 2025). This imbalance creates a “proinflammatory bias,” where the host is less capable of resolving inflammation (Bacher et al., 2014; Li et al., 2025). This failure of the resolution mechanisms leads to sustained increases in the levels of cytokines (IL-6, TNF-α, IL-17) that are capable of compromising the BBB, thereby facilitating the infiltration of peripheral immune mediators into the brain and influencing neural function (Bacher et al., 2014; Choi et al., 2016; Gładysz et al., 2018; Li et al., 2025).

In summary, fungal dysbiosis in ASD may act as more than a localized mucosal irritant. Through the activation of the Syk–CARD9–NF-κB axis and the NLRP3 inflammasome, and the subsequent skewing of the Th17/Treg balance, fungal dysbiosis appears to be associated with systemic inflammatory pathology. However, whether fungal dysbiosis serves as a primary driver or a secondary amplifier of inflammation in human ASD requires confirmation through prospective longitudinal studies and interventional trials. This cascade provides a mechanistic bridge explaining how gut-resident fungi can drive the neuroimmune alterations, specifically microglial activation and synaptic dysfunction, implicated in the pathogenesis of ASD.

#### 3.2.3 Neurotransmitter and metabolic disruptions

The human gut functions as a metabolic crucible where the resident microbiota synthesizes and modulates a vast array of neuroactive compounds (Caputi et al., 2021; Strandwitz, 2018). In the context of ASD, fungal dysbiosis—often co-occurring with bacterial imbalances—disrupts the production and homeostasis of these key metabolites (Averina et al., 2020; Pochakom et al., 2022). The resulting metabolic profile is characterized by the accumulation of neurotoxic byproducts and the depletion of neuroprotective factors. This section provides an in-depth analysis of the mechanistic pathways linking SCFAs, monoamine neurotransmitters, and fungal toxins to the neuropathology of ASD.

Critically, the disruption of these bacterial–fungal ecological dynamics, as detailed in section “2 Linking gut bacterial–fungal dysbiosis to systemic consequences,” fundamentally reshapes the metabolic landscape of the gut. As outlined previously, commensal bacteria are primary producers of key neuroactive metabolites, most notably SCFAs. The depletion of beneficial bacterial taxa, a hallmark of the dysbiotic state in NDDs, leads to a marked reduction in the production of metabolites like butyrate, which possesses neuroprotective and anti-inflammatory properties (Zheng et al., 2023). Concurrently, this bacterial dysbiosis erodes the competitive inhibition and immune-mediated containment that normally suppress fungal proliferation—a process ecologically defined as “niche release” (Gaspar et al., 2025; Severance et al., 2016). The resulting fungal expansion, particularly of opportunistic species like *Candida*, is not an isolated event but occurs within this altered metabolic milieu. Therefore, the effect of fungal dysbiosis within the context of this co-occurring bacterial dysbiosis and the resulting shifts in critical microbial metabolites must be examined to comprehensively understand the functional consequences of fungal dysbiosis. The following analysis of specific metabolites, including bacteria-derived SCFAs, is framed within this integrated pathophysiological model, wherein changes in the fungal community and the distorted metabolic output of the broader microbiota are inextricably linked drivers of pathology.

#### 3.2.4 Propionic acid (PPA) and hyperactivity

Elevated propionic acid (PPA) levels in ASD stool (Li et al., 2017; Wang et al., 2012) correlate with ASD-like phenotypes—hyperactivity, repetitive behaviors, and social deficits—following intraventricular administration in animal models. PPA crosses the BBB and enters neuronal cells (Chintharala et al., 2024). Once it enters cells, excess PPA accumulates in the mitochondria, where it sequesters carnitine to form propionyl-L-carnitine, leading to secondary carnitine deficiency (Fattorusso et al., 2019; Parellada et al., 2014; Thomas et al., 2010). This disruption impairs β-oxidation and the tricarboxylic acid (TCA) cycle, resulting in reduced adenosine triphosphate (ATP) production and altered neuronal energy metabolism (Fattorusso et al., 2019; Parellada et al., 2014; Thomas et al., 2010). Furthermore, PPA accumulation induces metabolic stress, generating reactive oxygen species (ROS) and depleting glutathione, which creates an oxidative environment that triggers the release of proinflammatory cytokines such as IL-6 and TNF-α (Thomas et al., 2010). These cytokines sensitize neuronal circuits and alter synaptic transmission, creating a foundation for neurobehavioral abnormalities (Benarroch, 2024; Vezzani and Viviani, 2015). Beyond these metabolic and oxidative stressors, PPA exerts direct and complex neuromodulatory effects that vary significantly with the kinetics of exposure. In chronic models involving direct central nervous system administration, such as repeated intracerebroventricular (ICV) infusions in rats, PPA modulates gap junction gating in regions like the striatum (MacFabe et al., 2007). This process is potentially driven by intracellular acidification, a consequence of the metabolic disruptions described above, which alters electrotonic coupling and calcium signaling (MacFabe et al., 2007). These changes are associated with increased neural excitability, as evidenced by epileptiform spiking in the hippocampus, cortex, and caudate nucleus (MacFabe et al., 2007). Such electrophysiological alterations coincide with specific behavioral abnormalities, including retropulsion, snake-like postures, turning, and limb dystonia, which contribute to the excitatory–inhibitory imbalance underpinning the hyperactivity and repetitive behaviors observed in ASD models (MacFabe et al., 2007). However, the neurochemical impact of PPA is not uniform and presents a dose-dependent dichotomy. In contrast to the excitatory profile of chronic exposure, acute systemic administration of PPA (e.g., intravenous injection) produces a divergent profile (Morland et al., 2018). Upon rapidly crossing the BBB, PPA is taken up by GABAergic neurons, where it acts as a potent inhibitor of mitochondrial GABA-T, with a *K_i_* of approximately 1 mmol/L (Morland et al., 2018). This enzymatic inhibition leads to a rapid accumulation of intracellular GABA, enhancing vesicular packaging and release (Morland et al., 2018). The resulting increase in synaptic and extrasynaptic GABAergic tone suppresses cerebral glucose metabolism and overall neuronal excitability, manifesting behaviourally as lethargy and reduced activity rather than hyperactivity (Morland et al., 2018). Ultimately, the net effect of PPA on the excitatory–inhibitory balance in ASD is determined by the specific parameters of exposure. While the chronic, low-grade elevation in PPA levels typical of gut dysbiosis aligns closely with the gap junction modulation and mitochondrial stress pathways that drive network excitability and hyperactive behaviors, the potential for acute GABAergic inhibition highlights the complexity of the role of PPA in the gut-brain axis.

#### 3.2.5 Butyrate, HDAC inhibition, and ASD-relevant gut-brain mechanisms

Butyrate is a major gut microbial metabolite that can act intracellularly as a HDAC inhibitor, with downstream effects on intestinal and systemic biology (Schulthess et al., 2019). In tissue-resident intestinal macrophages, butyrate induces an antimicrobial imprint that is predominantly linked to HDAC3 inhibition, rather than G-protein-coupled receptor (GPCR) signaling or broad suppression of cytokine transcription (Schulthess et al., 2019). This imprinting is associated with metabolic reprogramming that includes reduced mechanistic target of rapamycin (mTOR) activity and a shift toward altered glycolytic flux. This metabolic shift supports LC3-associated (microtubule-associated protein 1A/1B-light chain 3-associated) host defenses and the upregulation of antimicrobial effectors such as calprotectin (S100A8/S100A9) and other antimicrobial peptides (Schulthess et al., 2019). Importantly, the antimicrobial program can be activated with little or no increase in the levels of classical proinflammatory cytokines, indicating a decoupling of antimicrobial activity from overt mucosal inflammation in the gut mucosa (Schulthess et al., 2019). The HDAC-inhibitory action of butyrate is supported by studies showing that HDAC3 inhibition reproduces key antimicrobial phenotypes and that the effects of butyrate are diminished when HDAC3 expression is reduced or inhibited, placing HDAC3 upstream of the metabolic and antimicrobial reprogramming in macrophages (Schulthess et al., 2019). Chromatin-modifying changes consistent with HDAC inhibition include increased histone acetylation (e.g., Ac-H3 and Ac-H4) and coordinated transcriptional shifts toward antimicrobial/lysosomal programs, with limited increases in the levels of proinflammatory cytokines in this context (Schulthess et al., 2019).

Clinical metabolomic studies report reduced fecal butyrate levels and the depletion of key butyrate-producing taxa (e.g., *Ruminococcaceae*, *Eubacterium*, and *Lachnospiraceae*) in patients with ASD, suggesting diminished luminal butyrate signaling that could compromise intestinal barrier function and mucosal immunity, thereby influencing gut-brain axis signaling relevant to ASD phenotypes (Liu et al., 2019; Schulthess et al., 2019). This dysbiosis often involves a shift away from butyrate producers toward taxa associated with other SCFAs, potentially altering the balance of gut-derived immunometabolic signals that affect gut permeability and inflammation (Liu et al., 2019; Schulthess et al., 2019). The established mechanism by which butyrate, via HDAC3 inhibition, drives an antimicrobial and barrier-supportive program in macrophages without eliciting widespread proinflammatory cytokine production provides a plausible pathway through which ASD-associated reductions in butyrate levels could perturb local gut barrier integrity and immune homeostasis, thereby impacting gut-brain communication and neurodevelopmental trajectories (Chang et al., 2014; Liu et al., 2019; Schulthess et al., 2019).

#### 3.2.6 Alterations in serotonin and dopamine synthesis

Gastrointestinal serotonin synthesis is predominantly governed by enterochromaffin cells in the gut, and its magnitude is modulated by both tryptophan availability and microbial signals (Caspani et al., 2024). The current evidence consistently shows that changes in the gut microbiota in patients with ASD encompass not only bacterial dysbiosis but also alterations in the fungal component (mycobiota) (Yetgin, 2024). Although direct causal links between particular fungal taxa and central serotonin synthesis have not yet been established, fungus-derived signals may influence enteric serotonin production and peripheral 5-hydroxytryptamine (5-HT) levels indirectly through multiple pathways (Caspani et al., 2024). Fungal dysbiosis can, for example, impact gut barrier integrity, trigger mucosal immune responses with proinflammatory cytokines (IL-1β, IL-6, and TNF-α), and modulate tryptophan metabolism by affecting indoleamine 2,3-dioxygenase (IDO) activity, thereby shunting tryptophan away from serotonin synthesis toward the kynurenine pathway and altering the serotoninogenic milieu (Caspani et al., 2024; Kelly et al., 2017; Yetgin, 2024). Peripheral 5-HT levels in patients with ASD are inconsistent across studies. Since the 1970s, several reports have described hyperserotonaemia in patients with ASD (elevated 5-HT levels in whole blood or platelets) (Anderson et al., 1987; Fattorusso et al., 2019; Hanley et al., 1977; Minderaa et al., 1987), yet imaging studies suggest that the central serotonin synthesis capacity may be reduced during development in some children with ASD (hyposerotonemia) (Wang Q. et al., 2023). This apparent discordance highlights the complexity of the serotonergic system in individuals with ASD, potentially involving altered transport of the serotonin precursor (tryptophan) across the BBB, serotonin transporter (SERT) function, and genetic variations (e.g., SERT Ala56) that influence neuronal serotonin reuptake, as well as dysregulated feedback signaling from gut-derived serotonin to central circuits and the broader gut-brain feedback loops (Fattorusso et al., 2019; Kelly et al., 2017; Li et al., 2017; Wang Q. et al., 2023). Taken together, these factors imply that gut fungi are more likely to act as components of a broader, multikingdom regulatory milieu rather than as direct serotonin producers. Consistent with a multiomics framework, fungal dysbiosis interacts with co-occurring bacterial dysbiosis to reshape the gut metabolic landscape, including reductions in the abundance of beneficial SCFA-producing bacteria (e.g., butyrate producers), which may diminish the SCFA-driven stimulation of tryptophan hydroxylase 1 (TPH1) and constrain peripheral 5-HT production, thereby altering downstream serotonergic signaling implicated in ASD phenotypes (Fattorusso et al., 2019; Hadrich et al., 2024; Hughes and Ashwood, 2018; Kelly et al., 2017; Li et al., 2017, 2019; Strati et al., 2017; Wang Q. et al., 2023; Xiang et al., 2025). While these mechanistic inferences are biologically plausible and supported by associations among fungal composition shifts, immune activation, and SCFA metabolism, causal relationships remain to be established and should be validated in future studies using animal models, longitudinal designs, and interventional experiments.

With regard to dopamine, the existing literature suggests an indirect, ecosystem-level mode of regulation. Dysbiosis in individuals with ASD, including fungal overgrowth, may influence dopaminergic biosynthesis by affecting the host inflammatory state, the availability of precursors such as tyrosine, and the activity of key enzymes such as tyrosine hydroxylase (Fattorusso et al., 2019). Experimental models indicate that a chronic, gut-derived inflammatory environment can perturb dopaminergic circuits (Fattorusso et al., 2019). In addition, certain microbe-associated metabolites linked to dysbiosis may modulate dopaminergic signaling; for example, PPA has been shown to alter brain neurochemistry and mitochondrial function in animal models, potentially impacting dopamine pathways involved in rewards processing and motivation (Li et al., 2017; MacFabe et al., 2011). Although direct evidence demonstrating that gut fungal taxa regulate central dopamine synthesis in humans is limited, converging data from ASD microbiome studies imply that multikingdom interactions—bacteria and fungi—with host immune and metabolic responses may converge on the tyrosine–dopamine axis via inflammation, barrier integrity, and metabolite flux (Hadrich et al., 2024; Strati et al., 2017; Yetgin, 2024). Moreover, gut microbes [including fungi (Yetgin, 2024)] can itself produce neuroactive metabolites or neurotransmitter analogs (for instance, some bacteria produce dopamine precursors), although direct fungal evidence in this regard remains to be fully established (Li et al., 2017; Strandwitz, 2018; Wang Q. et al., 2023). Collectively, the current associative evidence supports a hypothetical model in which fungal dysbiosis may contribute to an altered gut-brain milieu that could indirectly influence dopamine synthesis in individuals with ASD, rather than acting as a direct neurotransmitter source. This proposed relationship requires validation through mechanistic studies and longitudinal observations. This interpretation awaits further validation of causality and the mechanism through integrated multiomics approaches, controlled animal models, and human interventional studies.

#### 3.2.7 Additional metabolic disruptions and fungal toxins

Beyond classical neurotransmitters, the gut mycobiome may contribute to the neurotoxic load in individuals with ASD through the production of specific metabolic byproducts. As previously described, the gut mycobiota in patients with ASD is often characterized by a well-documented increase in the abundance of *Candida* species, most consistently *Candida albicans* (Dargenio et al., 2023; Kantarcioglu et al., 2016; Zou et al., 2021). This fungal dysbiosis is notable because *Candida* species can release ammonia and other toxins, which have been hypothesized to contribute to autistic behaviors (Dargenio et al., 2023; Kantarcioglu et al., 2016; Li et al., 2017).

The finding of significantly higher fecal ammonia concentrations in children with ASD compared to controls provides a basis for proposing a role for ammonia in ASD. Elevated fecal ammonia levels may translate into higher systemic and brain ammonia levels, potentially contributing to neurotoxicity, but this hypothesis requires further investigation (Wang et al., 2012). Importantly, the elevated fecal ammonia level likely results from a combination of microbial sources and not solely from fungal dysbiosis. While *Candida* species, particularly *C. albicans*, which are frequently elevated in individuals with ASD, are hypothesized to release ammonia (Dargenio et al., 2023; Kantarcioglu et al., 2016; Wang et al., 2012; Zou et al., 2021), ammonia is also a major by-product of protein fermentation by gut bacteria (Wang et al., 2012). Furthermore, bacterial dysbiosis in patients with ASD often involves an increase in the abundance of specific bacteria such as *Clostridium* species, which are known producers of neurotoxic metabolites (Waligora-Dupriet et al., 2019). Therefore, the observed hyperammonaemia may stem from synergistic contributions of both bacterial and fungal dysbiosis. High colonic levels of ammonia have been shown to damage epithelial cells and increase gut permeability (Wang et al., 2012). Mechanistically, ammonia readily crosses the blood–brain barrier (Dasarathy et al., 2017). High brain ammonia levels (hyperammonaemia) are known to compromise astrocyte function (Dasarathy et al., 2017). A key neurotoxic mechanism involves the detoxification of ammonia in astrocytes, where it consumes α-ketoglutarate to generate glutamate and glutamine, leading to the depletion of tricarboxylic acid (Krebs) cycle intermediates and metabolic stress (Dasarathy et al., 2017). This mitochondrial dysfunction aligns with the broader concept of neuroinflammation. This neuroinflammatory state could be exacerbated by metabolic disturbances.

This neuroinflammatory state could be exacerbated by broader metabolic disturbances beyond the direct production of fungal toxins. Specifically, the intestinal dysbiosis characteristic of ASD extends to a disruption in the microbial synthesis of key neuroactive and immunomodulatory metabolites. This includes not only the previously discussed neurotransmitters and SCFAs, but also compounds such as GABA, biotin, and precursors to pivotal neuromodulators. Animal models of neurodevelopmental disorders support the link between gut dysbiosis and central neurotransmitter or synaptic dysfunction. For instance, studies using the maternal immune activation or BTBR mouse models of ASD have shown that gut dysbiosis leads to imbalances in tryptophan-derived metabolites (e.g., alterations in the serotonin and kynurenine pathways) and perturbs the glutamate-glutamine-GABA cycle, which are associated with behavioral abnormalities (Giuliano and Tognini, 2025; Wang X. et al., 2025). Furthermore, interventions such as fecal microbiota transplantation from patients to mice demonstrate that microbial alterations can directly influence behavior and associated neurochemical profiles (Wang X. et al., 2025). The intestinal fungal dysbiosis emphasized in this review likely exacerbates these deficits through converging pathways: by aggravating bacterial community disruption, it can compromise the bacterial microenvironment responsible for producing a balanced profile of neuroactive metabolites. For example, bacterial dysbiosis in ASD is linked to decreased production of neuroprotective metabolites like indole-3-propionic acid and kynurenic acid, while increasing pro-inflammatory molecules such as quinolinic acid (Giuliano and Tognini, 2025). Fungal dysbiosis, by driving bacterial ecological shifts, likely amplifies this imbalance. Thus, the impact of fungal dysbiosis converges mechanistically with broader neurotransmitter and synaptic abnormalities, operating within the “multihit” framework by disrupting the bacterial ecology essential for maintaining metabolic homeostasis.

Notably, current research cannot definitively determine a temporal sequence or a unidirectional causal relationship between mycobiota and bacteriobiota dysbiosis in individuals with ASD (Waligora-Dupriet et al., 2019). The existing evidence primarily indicates that these phenomena are frequently co-occurring and are likely interconnected, collectively contributing to the complex state of a destabilized gut ecosystem in affected individuals. The disturbance in bacterial communities (bacterial dysbiosis) may disrupt the microbial equilibrium, thereby fostering a permissive environment for fungal overgrowth (e.g., *Candida* spp.) (Waligora-Dupriet et al., 2019). Conversely, the proliferation of fungi may reciprocally alter the structure and function of the bacterial community. This bidirectional, potentially synergistic interaction underscores the complexity of multikingdom microbial dysregulation in ASD pathogenesis (Waligora-Dupriet et al., 2019).

Building upon the framework of bidirectional bacterial–fungal interactions, the pathophysiology of ASD can be further conceptualized as a “multihit” process (Parellada et al., 2014). This process is driven by a synergistic dysregulation of the multikingdom gut ecosystem, where fungal and bacterial dysbiosis are not sequential but co-occurring and mutually reinforcing (Parellada et al., 2014). In this model, fungal dysbiosis (e.g., *Candida* overgrowth) and bacterial dysbiosis (e.g., increased abundance of *Clostridium* species) collectively instigate a disrupted metabolic environment (Dargenio et al., 2023; Li et al., 2017; Strati et al., 2016; Waligora-Dupriet et al., 2019). While fungus-derived ammonia directly contributes to the neurotoxic load, its impact is synergistically magnified within a gut ecosystem concurrently reshaped by bacterial dysbiosis to favor the production of other harmful metabolites, such as PPA and phenolic compounds like *p*-cresol (Strati et al., 2016; Yetgin, 2024). Therefore, the interdependent dysregulation of both fungal and bacterial communities establishes a synergistic toxic environment in individuals with ASD. Concurrent exposure to elevated levels of PPA, ammonia, and *p*-cresol likely has compounded effects. Specifically, PPA-induced mitochondrial dysfunction and neuroinflammation could potentially render neurons more vulnerable to the excitotoxic effects associated with ammonia and the oxidative stress linked to other metabolites, illustrating the proposed “multihit” hypothesis in the context of ASD (Dasarathy et al., 2017; MacFabe et al., 2007; Parellada et al., 2014).

Critically, the neuropathological processes outlined in this “multihit” model—synaptic dysfunction, impaired neural circuit refinement, and neuroinflammation—are not exclusive to the core symptoms of ASD. They represent fundamental disruptions in brain development and function that are directly implicated in intellectual disability (ID), a condition that co-occurs in a significant subset of individuals with ASD and constitutes a major determinant of adaptive outcome (Alkuwaiti et al., 2025; Biagi et al., 2014). Therefore, the synergistic disruption of mitochondrial function, neuronal excitability, and neuroimmune homeostasis detailed above provides a plausible biological substrate for ID in the context of ASD. Specifically, disturbances in microbial-derived metabolites, such as reduced production of SCFAs like butyrate, may compromise epigenetic regulation of microglial and astrocyte function, processes critical for synaptic pruning and long-term potentiation (Han et al., 2025; Protic et al., 2025). In individuals with ASD and co-occurring ID, such multifactorial gut-brain axis dysregulation may be a key contributor to aberrant synaptic connectivity and the associated cognitive impairments.

### 3.3 Conclusion: a multihit hypothesis for mycobiota-driven pathogenesis in ASD

Emerging associative evidence implicates an altered gut mycobiota composition in ASD pathophysiology, characterized by fungal dysbiosis and its potential interaction with the gut-brain axis, though causal directionality remains to be established (Yetgin, 2024). Studies have revealed distinct fungal community structures in patients with ASD, with an increased prevalence of specific *Candida* species and alterations in commensal fungal populations (Strati et al., 2017). Mechanistically, fungal dysbiosis may compromise intestinal barrier integrity, exacerbate systemic immune dysregulation via inflammatory signaling cascades, and disrupt neurotransmitter and metabolic homeostasis through the production of neuroactive compounds and toxins (Yetgin, 2024). These multifaceted interactions within the gut ecosystem highlight the complex interplay between fungal and bacterial communities, which potentially contribute to neuroinflammation and neurobehavioral manifestations observed in patients with ASD (Yetgin, 2024). Further research is warranted to elucidate causal relationships and therapeutic implications of mycobiota alterations in individuals with ASD.

## 4 Gut mycobiota dysbiosis in patients with ADHD

### 4.1 Evidence of an altered fungal composition in patients with ADHD

Case-control and birth cohort studies demonstrate altered gut mycobiome patterns in ADHD patients that may influence neurodevelopment through gut-brain axis pathways (Wang L. J. et al., 2023). Cross-sectional analyses of pediatric samples show ADHD is associated with higher *Ascomycota* and lower *Basidiomycota* abundance, indicating fungal community structure shifts rather than overall richness changes (Wang L. J. et al., 2023). At the genus level, increased *Candida*—particularly *C. albicans*—is frequently observed and linked to elevated intestinal permeability in vitro, suggesting a mechanism for fungal dysbiosis contributing to systemic inflammation and neurodevelopmental perturbations (Wang L. J. et al., 2023). Beta-diversity analyses consistently show a distinct mycobiome composition between patients with ADHD and neurotypical controls, while alpha-diversity often shows little or variable differences, implying that ADHD-associated differences reflect compositional remodeling rather than extensive losses or gains in fungal diversity (Wang L. J. et al., 2023). Longitudinal studies of infants have provided converging, though still preliminary, evidence that early-life fungal signatures (e.g., *Candida*, *Rhodotorula*, *Malassezia*, and *Saccharomyces* OTUs) correlate with a later ADHD risk, supporting the hypothesis that early gut colonization patterns may influence neurodevelopmental trajectories (Cassidy-Bushrow et al., 2023). Collectively, these findings from multiple cohorts and methodologies (ITS sequencing, ITS1/ITS2 analysis, and 18S rRNA approaches) point to a reproducible pattern of gut mycobiome alterations in individuals with ADHD that may perturb gut barrier function and immune signaling, thereby contributing to gut-brain axis-mediated neurodevelopmental outcomes (Wang L. J. et al., 2023). However, the results vary by cohort, age, sequencing target, and analytical approach, underscoring the need for standardized protocols, larger longitudinal samples, and mechanistic studies to clarify causality and therapeutic implications (Checa-Ros et al., 2021). Potential confounders such as diet, antibiotic exposure, delivery mode, sex, and BMI have been considered in several studies, and multivariate analyses commonly adjust for these factors to isolate relevant fungal signals (Cassidy-Bushrow et al., 2023).

### 4.2 Mechanistic insights underlying fungal dysbiosis in patients with ADHD

#### 4.2.1 Effect of a fungal imbalance on the intestinal mucosal barrier

Enteric fungal dysbiosis in individuals with ADHD is characterized by a notable enrichment of *Candida* species, most consistently *Candida albicans*, accompanied by phylum-level shifts favoring *Ascomycota* and a relative reduction in the abundance of *Basidiomycota* (Hadrich et al., 2024; Wang L. J. et al., 2023). This fungal imbalance is plausibly implicated in compromising intestinal mucosal barrier integrity, thereby contributing to a state of increased intestinal permeability known as a “leaky gut” (Hadrich et al., 2024; Iliev and Cadwell, 2021; Lionakis et al., 2023; Wang L. J. et al., 2023). *In vitro* data using Caco-2 cell monolayers indicate that filtrates or secreted products from *C. albicans* substantially increase epithelial barrier permeability, as evidenced by increased dye leakage and increased transepithelial flux of serum components (Wang L. J. et al., 2023). These findings imply that fungal secretions can disrupt the tight junction architecture and epithelial cytoskeleton, potentially via direct interference with tight junction proteins (e.g., occludin, claudins, and ZO-1) or through signaling cascades that drive cytoskeletal remodeling (Wang L. J. et al., 2023). Such barrier perturbations plausibly permit translocation of luminal MAMPs and fungal metabolites into the portal and systemic circulation, increasing the exposure of immune cells and hepatic surveillance systems to pathogenic fungal components (Leonardi et al., 2022; Lionakis et al., 2023; Wang L. J. et al., 2023). The consequence of this barrier breach is the systemic dissemination of fungus-derived molecules, such as β-glucans, and other metabolites (Lionakis et al., 2023). These circulating fungal ligands can serve as PAMPs that engage innate and adaptive immune receptors, eliciting a cascade of immune activation (Lionakis et al., 2023). Preclinical studies, primarily in mouse models, suggest a recurring theme of C-type lectin receptors (CLRs) such as Dectin-1, Dectin-2, and Dectin-3 on antigen-presenting cells recognizing fungal components (Iliev and Cadwell, 2021; Lionakis et al., 2023). In these models, receptor engagement triggers downstream signaling through Syk kinase and the CARD9–MALT1–BCL-10 axis, culminating in NF-κB activation and production of proinflammatory cytokines like IL-6, IL-23, and TNF-α (Iliev and Cadwell, 2021; Lionakis et al., 2023). In mouse models, this inflammatory milieu promotes the differentiation and expansion of CD4^+^ Th17 cells, along with the concomitant secretion of IL-17A and IL-22 (Leonardi et al., 2022; Lionakis et al., 2023). Studies in mice indicate that IL-22 contributes to mucosal defenses and epithelial renewal, whereas IL-17A exerts robust proinflammatory effects, including neutrophil recruitment (Leonardi et al., 2022; Lionakis et al., 2023). In the context of persistent fungal dysbiosis, a maladaptive Th17/IL-17 signaling balance is proposed to contribute to tissue injury and sustained barrier disruption (Leonardi et al., 2022; Lionakis et al., 2023).

Concurrent with barrier and immune mechanisms, fungal dysbiosis interacts with bacterial community dynamics to influence barrier integrity and neuroimmune signaling (Iliev and Cadwell, 2021). Preclinical evidence from mouse models suggests that certain bacterial taxa with barrier-supporting functions, such as certain *Bacteroides* spp. and *Blautia* spp., may promote barrier fortification through mechanisms like hypoxia-inducible factor (HIF-1α)-driven pathways and antimicrobial peptide production [e.g., cathelicidin-related antimicrobial peptide (LL-37)], thereby potentially restraining *Candida* colonization and stabilizing the mucosal milieu (Lionakis et al., 2023). Conversely, the overgrowth of *Candida* can perturb the bacterial community structure via resource competition, pH alterations, or the secretion of antifungal or antibiosis factors, potentially diminishing beneficial bacterial collaborators that support barrier integrity (Dalle et al., 2010; Deveau et al., 2018; Gaspar et al., 2025; Hadrich et al., 2024; Shuai et al., 2022; Strati et al., 2017; van Tilburg Bernardes et al., 2020). Interkingdom crosstalk thus represents a bidirectional regulatory axis in which expanded fungal communities and bacterial dysbiosis mutually reinforce each other, shaping the inflammatory tone and the pool of microbial metabolites, including SCFAs, which themselves modulate mucosal immunity and CNS function (van Tilburg Bernardes et al., 2020).

#### 4.2.2 Immune activation and systemic inflammation: from barrier breach to neuroimmune signaling pathways

Building on the barrier disruption detailed in section “4.2.1 Effect of a fungal imbalance on the intestinal mucosal barrier,” fungal components such as β-glucans enter the circulation and engage C-type lectin receptors (e.g., Dectin-1/2/3) on immune cells (Lionakis et al., 2023). This interaction activates the Syk–CARD9–NF-κB axis, driving proinflammatory cytokine production (IL-6, IL-23, IL-1β, TNF−α) as described in section “3.2.2 Immune dysregulation and systemic inflammation” (Lionakis et al., 2023). Th17-derived IL-17A amplifies inflammation. Under persistent fungal exposure, this response shifts from protective to pathological, driving excessive systemic inflammation (Iliev and Cadwell, 2021; Leonardi et al., 2022; Lionakis et al., 2023; Zhang et al., 2025). Concurrently, the sustained inflammatory milieu further compromises epithelial barrier function. The disruption of barrier integrity, in turn, creates favorable conditions for fungal colonization and antigen exposure, which perpetuate the stimulation of Th17 immunity. This process establishes a self-amplifying, feed-forward loop that exacerbates both systemic inflammation and barrier impairment (Iliev and Cadwell, 2021; Leonardi et al., 2022; Lionakis et al., 2023; Zhang et al., 2025).

These translocated fungal ligands activate innate immune surveillance in hepatic and splenic networks (Leonardi et al., 2022; Lionakis et al., 2023). Human studies in ADHD cohorts link Candida enrichment to elevated systemic inflammatory markers and barrier dysfunction, supporting a gut–immune–brain axis despite causal limitations (Hadrich et al., 2024; Wang L. J. et al., 2023). IL-17A may act as a neuromodulator: rodent studies show fungus-induced IL-17A can influence social and reward-related behaviors via neuronal IL-17 receptors (Leonardi et al., 2022; Lionakis et al., 2023). However, translation to humans remains inferential and requires longitudinal confirmation.

Fungal overgrowth disrupts bacterial consortia, altering SCFA profiles and exacerbating inflammation (Leonardi et al., 2022; van Tilburg Bernardes et al., 2020). This crosstalk amplifies systemic inflammatory signals reaching the CNS via humoral (cytokines crossing BBB) and neural (vagal afferents) routes (Hadrich et al., 2024; Leonardi et al., 2022; Lewis et al., 2025), potentially modulating monoaminergic neurotransmission and executive functions relevant to ADHD (Checa-Ros et al., 2021).

#### 4.2.3 CNS neurotransmitter dysregulation: from cytokine signaling to neural circuit disruption

These potential modulations of key neurotransmitter systems occur through specific mechanisms by which peripherally derived immune signals interface with the CNS. Proinflammatory cytokines such as IL-6, IL-1β, and TNF-α can access the CNS via several routes, including direct transit across a permeable or compromised BBB and signaling through circumventricular organs where the BBB is inherently leaky (Lewis et al., 2025). Within the CNS, these cytokines can modulate the synthesis, release, reuptake, and receptor sensitivity of monoamines—dopamine, norepinephrine, and serotonin—in prefrontal and subcortical networks critical for attention, working memory, and executive control (Lewis et al., 2025). For instance, IL-6 can influence tryptophan metabolism *via* the tryptophan 2,3-dioxygenase (TDO) and indoleamine 2,3-dioxygenase (IDO) pathways, potentially reducing central serotonin availability and generating neuroactive kynurenine metabolites that affect glutamatergic signaling and microglial activation (Checa-Ros et al., 2021; Lewis et al., 2025). TNF-α and IL-1β can alter dopamine transporter function and dopaminergic neuron firing in frontostriatal circuits, potentially shifting the balance between tonic and phasic dopamine signaling that underpins sustained attention and cognitive control (Borrajo et al., 2014; Channer et al., 2023; Felger and Lotrich, 2013; Goldsmith et al., 2023; Lee et al., 2018; Lewis et al., 2025; Lewitus et al., 2014; Miller et al., 2017). IL-1β can also modulate norepinephrine signaling in the prefrontal cortex, influencing the signal-to-noise ratio and executive performance (Lee et al., 2018). Collectively, these cytokine-driven changes can recalibrate frontostriatal dynamics and rewards processing, contributing to ADHD-related impairments in attention, working memory, inhibitory control, and susceptibility to distraction.

Following the modulation of key neurotransmitter systems, these peripherally derived immune signals extend their influence to synaptic plasticity and large-scale neural networks. Systemic inflammation can prime microglia, altering synaptic pruning, receptor expression, and dendritic spine dynamics within prefrontal and striatal circuits (Lewitus et al., 2014; Miller et al., 2017). These changes lead to desynchronized thalamocortical and cortico-striatal communication, disrupting the maturation and efficiency of critical networks like the default mode network and frontoparietal control systems, and thereby contributing to deficits in planning, cognitive flexibility, and response inhibition that are characteristics of ADHD (Bush, 2010; Checa-Ros et al., 2021; Goldsmith et al., 2023; Konrad and Eickhoff, 2010; McCarthy et al., 2014; Rubia et al., 2019). Concurrently, the integrity of the blood–brain barrier can be compromised by inflammatory mediators, facilitating the central entry of peripheral immune signals and altering the chemical milieu in the CNS via changes in tight junction proteins and transporter function (Channer et al., 2023; Felger and Lotrich, 2013; Goldsmith et al., 2023). This process further modulates monoamine availability in key regions such as the dorsolateral prefrontal cortex and anterior cingulate cortex (Boyle et al., 2024; Bush, 2010; Cools and Arnsten, 2022; Felger and Lotrich, 2013; Goldsmith et al., 2023; Lee et al., 2018; Miller et al., 2017). The peripheral inflammatory tone also interacts with gut-derived metabolites and vagal signaling. SCFAs and tryptophan-derived metabolites can influence CNS function via afferent vagal pathways, which in turn can rapidly adjust monoaminergic signalling and plasticity in attention and rewards networks, potentially exacerbating or mitigating ADHD-related symptoms (Checa-Ros et al., 2021; Lewis et al., 2025).

Collectively, the multi-level disruptions in neurotransmitter signaling, synaptic plasticity, and peripheral-CNS communication described above converge to compromise the integrated functioning of large-scale brain networks. The resultant inefficiency in frontostriatal and cortical networks directly undermines the cognitive domains of working memory, cognitive flexibility, and inhibitory control (Alkuwaiti et al., 2025). These executive functions constitute core pillars of general intellectual functioning, thereby linking the proposed gut-derived dysregulation to the cognitive deficits that are a hallmark of ADHD and can manifest as ID in more severe or co-morbid presentations (Alkuwaiti et al., 2025; Ramírez et al., 2022). Mechanistically, an altered gut microbiota can affect the availability of dopamine precursors (e.g., phenylalanine and tyrosine) and shift the balance of kynurenine pathway metabolites, both of which are critical for modulating prefrontal cortex-mediated executive processes (Alkuwaiti et al., 2025). Furthermore, a reduction in beneficial bacteria such as *Faecalibacterium prausnitzii*, which is associated with a pro-inflammatory state, may impair neuronal energy metabolism by diminishing mitochondrial support normally provided by microbial short-chain fatty acids, thereby exacerbating cognitive impairment (Protic et al., 2025).

#### 4.2.4 Integration and translational caveats

In summary, an integrative, testable cascade can be proposed: intestinal fungal dysbiosis may lead to barrier disruption and the translocation of fungal components, which could trigger CLR–Syk–CARD9–NF-κB-mediated pro-inflammatory cytokine production and Th17/IL-17 response. This proposed mechanistic framework is grounded in well-established immunological pathways; however, its specific application to ADHD pathophysiology is primarily supported by associative human data (Leonardi et al., 2022; Lionakis et al., 2023). This process results in systemic inflammation whose signals, which are conveyed by humoral and neuronal pathways, promote CNS neuroinflammation, alter monoaminergic neurotransmission and synaptic plasticity in frontostriatal circuits, and ultimately yield neurobehavioral deficits consistent with the core symptoms of ADHD (Channer et al., 2023; Lee et al., 2018; Lewis et al., 2025). Notwithstanding this integrative cascade, establishing causality in humans remains challenging given the predominantly cross-sectional data, heterogeneity in mycobiome assessment methods, age ranges, ADHD subtypes, and medication statuses (Cassidy-Bushrow et al., 2023; Checa-Ros et al., 2021; Hadrich et al., 2024; Lewis et al., 2025; Wang L. J. et al., 2023). Furthermore, the interkingdom context adds another layer of complexity, as protective bacterial taxa can temper Th17 responses and promote anti-inflammatory SCFA production, whereas fungal overgrowth can tilt the balance toward proinflammatory signaling; disentangling direct fungal effects from those mediated by bacterial dysbiosis and metabolite shifts is therefore essential (Hadrich et al., 2024; Iliev and Cadwell, 2021; Lewis et al., 2025; Lionakis et al., 2023; van Tilburg Bernardes et al., 2020). While animal work provides a coherent mechanistic link between fungal IL-17 signaling and neural outcomes, human confirmation is still associative, and longitudinal multiomics studies are needed to establish temporality and causality and to delineate ADHD-specific neurobiological readouts. Thus, while the proposed mechanistic framework is grounded in well-established immunological pathways, such as the CLR–Syk–CARD9–NF-κB signaling axis, its specific application to ADHD pathophysiology is primarily supported by associative human data (e.g., studies linking *Candida*-dominant mycobiomes to intestinal permeability) (Lionakis et al., 2023; Wang L. J. et al., 2023). Therefore, in areas where direct human ADHD evidence is limited, the claims should be framed as plausible links awaiting causal substantiation, with an explicit acknowledgment of methodological heterogeneity and translational gaps (Cassidy-Bushrow et al., 2023; Checa-Ros et al., 2021; Lewis et al., 2025; Lionakis et al., 2023; Wang L. J. et al., 2023).

### 4.3 Conclusion: charting future research on fungal dysbiosis in patients with ADHD

In conclusion, while preclinical models and associative human data suggest a compelling link between fungal dysbiosis, systemic inflammation, and ADHD-related neurobehavioral deficits, further research is crucial. Longitudinal, multiomics studies incorporating advanced mycobiome profiling, detailed immunophenotyping, and neuroimaging are needed to establish causality, delineate the specific contributions of fungal and bacterial communities, and identify potential therapeutic targets within this complex gut-immune-brain axis.

## 5 Gut mycobiota dysbiosis in patients with RTT

### 5.1 Evidence of an altered fungal composition in RTT patients

Shifting focus to RTT, a neurodevelopmental disorder with well-defined monogenic origin, investigations have revealed profound gut mycobiome dysbiosis (Strati et al., 2016). This dysbiosis is characterized by reduced fungal diversity and drastically altered composition distinct from healthy controls (Strati et al., 2016). The most prominent alteration across studies is significant enrichment of opportunistic fungi from the genus Candida, which dominates fecal fungal communities in RTT patients (Strati et al., 2016). Notably, species-level analysis has isolated *Candida* parapsilosis strains with virulent traits from RTT intestines (Strati et al., 2016), consistent with the profound fungal dysbiosis described in this condition (Chin et al., 2020; Yetgin, 2024). Conversely, this expansion of *Candida* occurs alongside a depletion of beneficial or commensal fungal taxa (Yetgin, 2024). According to comparative analyses, the gut mycobiota of subjects with RTT displays a marked reduction in the relative abundance of *Saccharomyces* species (e.g., *S. cerevisiae*), which are commonly associated with a healthy gut profile and possess immunomodulatory properties (Hadrich et al., 2024; Yetgin, 2024). Similarly, the abundance of other beneficial genera such as *Penicillium* appears to be decreased in this specific clinical context (Strati et al., 2016). The common gut commensal eukaryote of the protozoan genus *Blastocystis*, which is frequently detected in healthy controls, was largely absent in the RTT cohort (Strati et al., 2016), indicating that gut eukaryotic dysbiosis in RTT extends beyond fungi. These specific changes, namely the increased abundance of potentially pathogenic *Candida* and the loss of beneficial fungi like *Saccharomyces*, are understood to reflect a dysbiotic ecosystem adapted to the costive gastrointestinal niche of RTT (Strati et al., 2016). The evidence for these findings is primarily derived from culture-independent, high-throughput DNA sequencing targeting the fungal ITS region, combined with bioinformatics and statistical analyses to robustly compare the mycobiota composition and identify discriminant taxa.

### 5.2 Mechanistic insights underlying fungal dysbiosis in patients with RTT

#### 5.2.1 Effect of an MeCP2 impairment on the gut environment

The pivotal genetic defect in RTT, loss-of-function mutations in the methyl-CpG-binding protein 2 (*MeCP2)* gene, extends beyond the central nervous system to fundamentally alter the gastrointestinal environment, thereby creating conditions conducive to microbial dysbiosis (Strati et al., 2016). *MeCP2* has been shown to be a critical regulator of several interconnected systems within the gut, and its impairment contributes to a pro-dysbiotic niche through three primary mechanisms: disruption of the enteric nervous system (ENS), compromise of the mucosal barrier, and dysregulation of neuroimmune pathways (Cordone, 2024; Strati et al., 2016). Specifically, *MeCP2* is expressed in the ENS, and its dysfunction is directly implicated in the disordered gut motility characteristic of RTT, leading to diminished gastrointestinal regulation, an increased intestinal transit time, and chronic constipation—a symptom that has been reported in more than 70% of RTT patients (Cordone, 2024; Strati et al., 2016). Consequently, this costive gut environment alters microbial colonization dynamics by extending the retention of luminal contents, which favors the proliferation of a dysbiotic microbial community adapted to these conditions (Cordone, 2024). Importantly, recent evidence indicates that disruption of the gut microbiome, particularly the depletion of butyrate-producing *Clostridia* species, reduces butyrate production leading to epithelial oxygenation, thereby disrupting the mucosal hypoxic environment that normally limits *Candida* growth and promoting *C. albicans* expansion (Deveau et al., 2018; Marsaux et al., 2025; Mishra and Koh, 2024). This mechanism is particularly relevant in the context of RTT-associated dysbiosis, where alterations in bacterial community composition may compromise the anaerobic conditions that maintain colonization resistance against facultative anaerobic fungi. Furthermore, the integrity of the gut’s mucosal barrier is compromised, as impairments in *MeCP2* function are hypothesized to affect the regulation of genes essential for maintaining the protective mucus layer secreted by goblet cells (Cordone, 2024; Strati et al., 2016). These changes can lead to a reduction in the mucin layer, weakening the physical barrier that segregates luminal microbes from the host epithelium and predisposing the gut to colonization by opportunistic pathobionts, such as *Candida* and *Escherichia/Shigella*, whose translocation contributes to systemic inflammation (Strati et al., 2016, 2018; Yadav et al., 2024). Notably, fungal pathogens exhibit distinct colonization advantages in this compromised mucosal environment. *Candida* species express adhesins (such as Als proteins) that facilitate adherence to exposed epithelial surfaces when the mucin barrier is diminished (Liu et al., 2025). Moreover, the thinning of the mucus layer reduces the physical barrier that separates luminal fungi from the host epithelium. Importantly, mucins and mucin O-glycans within the mucus layer act as natural inhibitors of *Candida* albicans pathogenicity by suppressing filamentation-associated gene expression and blocking the yeast-to-hyphal transition (Liu et al., 2025; Takagi et al., 2022). Therefore, mucus layer depletion not only facilitates fungal access to epithelial surfaces but also removes the suppressive effects of mucin glycans on fungal morphological switching, thereby promoting the transition from commensal yeast forms to invasive hyphal forms—a transition critical for tissue invasion and pathogenicity (Liu et al., 2025; Takagi et al., 2022). The compromised barrier also permits bidirectional exchange of metabolites and signaling molecules between fungi and host immune cells, potentially accelerating the transition from mutualistic fungal colonization to pathogenic overgrowth (Allert et al., 2018; Sprague et al., 2024).

Lastly, *MeCP2* mutations disrupt the tightly regulated neuroimmune network, causing an inadequate or dysregulated immune response at the mucosal surface (Cordone, 2024). *MeCP2* deficiency has been shown to influence cytokine regulation, affecting Th1/Th17 and Treg cell balance (Gładysz et al., 2018; Hernández-Santos and Gaffen, 2012; Leoncini et al., 2015; Strati et al., 2016). The dysregulated immune environment in RTT is characterized by altered Th1/Th2/Th17 responses, with elevated pro-inflammatory cytokines contributing to a subclinical inflammatory state (Gładysz et al., 2018; Leoncini et al., 2015; Strati et al., 2016). While Th17 cells and their cytokine products (IL-17 and IL-22) are essential for maintaining epithelial barrier integrity and coordinating antifungal immunity, the precise impact of *MeCP2* deficiency on these specific antifungal defense mechanisms in RTT remains to be fully elucidated (Aggor et al., 2020; De Luca et al., 2010; Napoli et al., 2025; Zelante et al., 2009). Furthermore, *MeCP2* deficiency-linked immune dysregulation could potentially compromise antifungal effector pathways (including those dependent on neutrophil activity), thereby diminishing fungal clearance and permitting persistent colonization by *Candida* and other fungal taxa (Caputi et al., 2024; Gonçalez et al., 2024; Sparber and LeibundGut-Landmann, 2019; Strati et al., 2016; Zelante et al., 2009). To date, no studies have directly and rigorously demonstrated a causal relationship between *MeCP2* deficiency and impaired neutrophil phagocytosis, killing, or fungal clearance activity, representing an important area for future investigation.

#### 5.2.2 Bacterial dysbiosis as a precursor to fungal overgrowth

The intimate coexistence of bacterial and fungal communities suggests that perturbations in the bacteriome can significantly influence the mycobiome (Strati et al., 2016). In patients with RTT, the characteristic loss of bacterial diversity, often involving a decrease in the abundance beneficial commensals and an overabundance of potentially proinflammatory taxa, is a key factor contributing to fungal dysbiosis (Strati et al., 2016). This process unfolds through several interconnected mechanisms. First, the reduced abundance of a healthy commensal population compromises competitive exclusion, thereby freeing up essential nutrients, such as simple carbohydrates, and physical adhesion sites on the intestinal mucosa that fungi like *Candida* can exploit (Iliev and Leonardi, 2017). Second, the depletion of specific bacterial taxa, such as *Lactobacillus* and *Bifidobacterium*, diminishes the direct inhibition of fungal growth (Strati et al., 2016). This loss is critical, as these bacteria are known to produce antifungal compounds, including bacteriocins and SCFAs, that can disrupt fungal cell membranes or interfere with quorum-sensing signals necessary for virulence (Iliev and Leonardi, 2017). Consequently, the RTT-associated alterations in these populations create a more permissive environment. Furthermore, the altered metabolic output of the dysbiotic community can actively support fungal proliferation through metabolic crosstalk (Zhang et al., 2022). For instance, shifts in bacterial fermentation can result in the production of metabolites like specific amino acids or alcohols that serve as alternative nutrient sources for *Candida*, or they can alter local environmental conditions, such as pH and oxygen tension, to favor fungal growth and morphogenesis (Zhang et al., 2022). Collectively, the erosion of bacterial competition, the loss of direct antifungal inhibition, and the establishment of a supportive metabolic niche create conditions that facilitate the overgrowth of opportunistic fungi such as *Candida* (Iliev and Leonardi, 2017; Jiang et al., 2020; Yadav et al., 2024).

#### 5.2.3 Roles of SCFAs in dysbiosis and gut barrier dysfunction

A key metabolic feature of the gut environment of patients with RTT is the abnormal accumulation of specific SCFAs, with studies consistently showing significantly elevated fecal levels of propionate, isobutyrate, and isovalerate in affected individuals (Borghi and Vignoli, 2019; Cordone, 2024; Strati et al., 2016). These dysregulated microbial metabolites are implicated as key contributors to local gut pathophysiology through two interconnected mechanisms. First, high concentrations of SCFAs are known to inhibit colonic motility presumably through mechanisms such as the SCFA-mediated release of peptide YY (PYY) from enteroendocrine cells, which in turn suppresses smooth muscle contractions (Strati et al., 2016). Consequently, this impaired motility contributes directly to the chronic constipation prevalent in patients with RTT, prolonging the intestinal transit time and thereby reinforcing the dysbiotic microbial community by extending its exposure to retained luminal contents (Cordone, 2024; Strati et al., 2016). Second, these elevated SCFA levels can disrupt the integrity of the mucosal barrier by interfering with the normal exocytosis of mucins from goblet cells (Strati et al., 2016). This compromise in barrier function leads to a reduction in the protective mucin layer, which facilitates the translocation of pathobionts like *Candida* and *Escherichia/Shigella* across the epithelium and contributes to the low-grade systemic inflammation observed in patients with RTT (Strati et al., 2016; Yadav et al., 2024). Beyond the gut, these SCFAs exert broad systemic effects that may intersect with the neurodevelopmental pathology of RTT (Borghi and Vignoli, 2019). As signaling molecules, SCFAs, particularly propionate and butyrate, can modulate host gene expression (potentially via HDAC inhibition), influence neurotransmitter systems, and affect mitochondrial function (MacFabe, 2015; Nankova et al., 2014; Strati et al., 2016). Therefore, the altered SCFA profile in individuals with RTT may represent a critical mechanistic link between gut dysbiosis and the systemic manifestations of the disorder, including both gastrointestinal dysfunction and potential contributions to neuroinflammation and behavioral phenotypes.

#### 5.2.4 Mucosal immune responses and their contributions to fungal pathogenesis

A compromised mucosal immune response in individuals with RTT represents a critical factor in the shift from fungal commensalism to pathogenic overgrowth (Strati et al., 2018). This immune dysfunction manifests as a subclinical inflammatory status within the gastrointestinal tract, as evidenced by elevated levels inflammatory markers, including fecal calprotectin, erythrocyte sedimentation rate (ESR), and serum IgA (Strati et al., 2016). This low-grade inflammation is likely instigated by the translocation of microbial components through a compromised mucosal barrier, which in turn triggers a dysregulated immune response (Cordone, 2024). The underlying genetic defect in RTT directly impacts immune function, as *MeCP2* is a known epigenetic regulator of immune and inflammatory responses that plays a critical role in the differentiation and function of Th cells (Strati et al., 2018). Specifically, *MeCP2* deficiency is implicated in the impaired regulation of Th1 and Th17 cell lineages—pathways essential for mounting an effective antifungal defense at mucosal surfaces (Strati et al., 2018). Consequently, the mucosal immune system is less capable of controlling fungal populations (Strati et al., 2018). This impairment is compounded by aberrant cytokine profiles; for instance, while innate immune cells produce elevated levels of pro-inflammatory cytokines like IL-1β in response to *Candida* isolates from RTT patients, they also induce the production of the immunosuppressive cytokine IL-10 at significantly higher levels (Strati et al., 2018). Such a dysregulated cytokine environment, characterized by non-resolving inflammation, can impair fungal clearance and promote microbial persistence (Strati et al., 2018). In this permissive niche, opportunistic fungi like *Candida parapsilosis* can transition to pathogenic forms, exhibiting enhanced virulence-related traits such as increased biofilm formation and resistance to antifungals, thereby perpetuating the cycle of inflammation and dysbiosis characteristic of RTT (Cordone, 2024; Strati et al., 2018).

### 5.3 Conclusion

**FIGURE 1:**
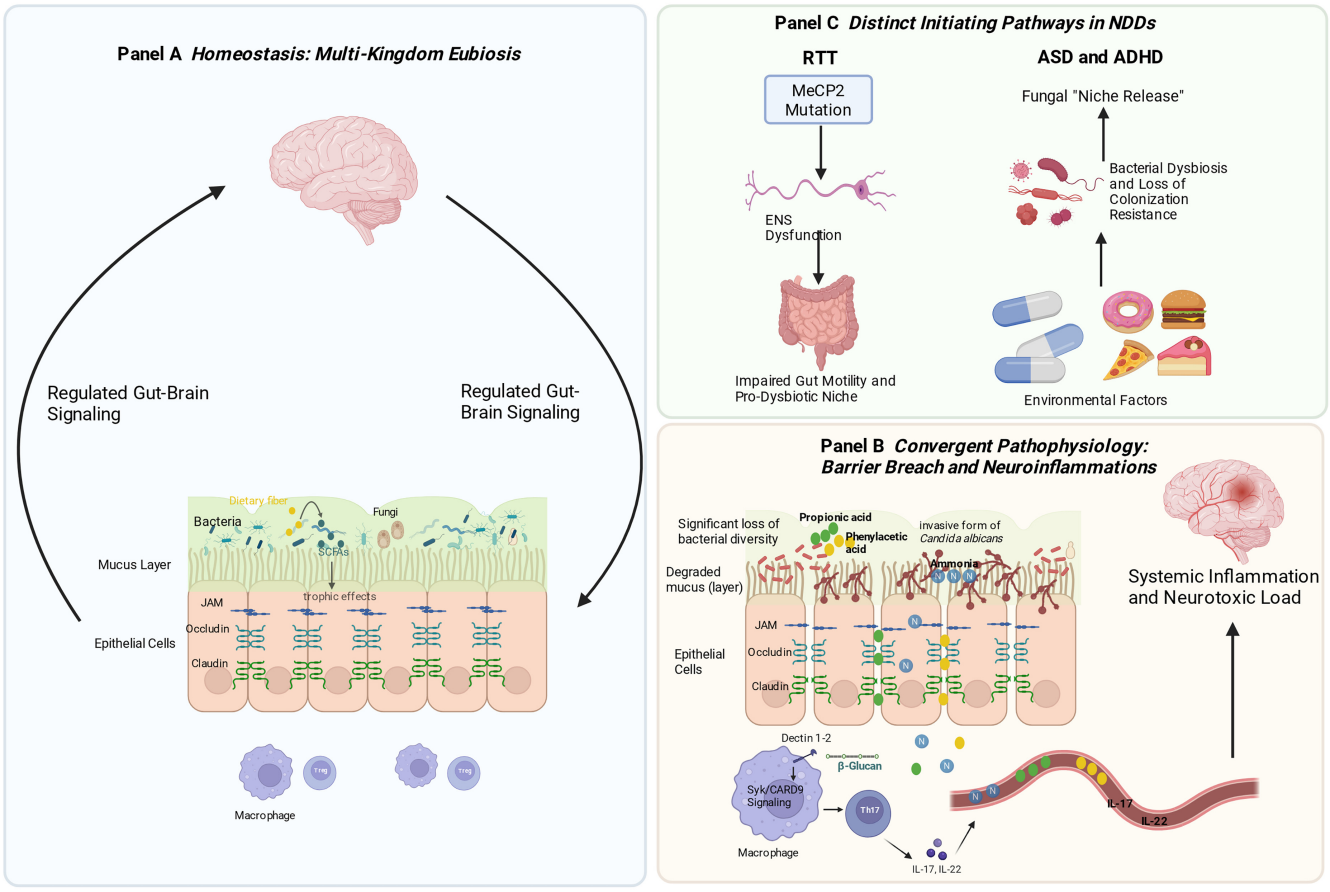
The gut mycobiota in neurodevelopmental disorders: from homeostasis to convergent pathophysiology via distinct initiating pathways. **(A)** Illustrates multi-kingdom eubiosis under homeostatic conditions, where bacterial colonization resistance maintains fungal commensalism and regulated gut-brain signaling. **(B)** Depicts the convergent pathophysiology characterized by barrier breach, bacterial diversity loss, fungal overgrowth (*Candida* invasion), and neuroinflammation via the Dectin-1/Syk/CARD9/Th17 axis. **(C)** Contrasts the distinct initiating mechanisms: the “top-down” pathway in RTT (MeCP2 mutation → ENS dysfunction → pro-dysbiotic niche) versus the “bottom-up” pathway in ASD/ADHD (environmental factors → bacterial dysbiosis → fungal niche release).

In summary, the gastrointestinal pathology in patients with RTT is sustained by a series of interconnected feedback loops that lock the gut into a stable dysbiotic and proinflammatory state. The core driver is the constipation–dysbiosis cycle initiated by MeCP2 deficiency in the enteric nervous system, which impairs gut motility (Strati et al., 2016). This cycle leads to luminal stagnation, promoting microbial overgrowth and the excessive production of specific SCFAs, notably, propionate, isobutyrate, and isovalerate (Cordone, 2024; Strati et al., 2016). These SCFAs further suppress smooth muscle contraction through mechanisms such as PYY release, thereby reinforcing constipation and closing the first feedback loop (Cordone, 2024; Strati et al., 2016). This cycle subsequently drives a barrier–immune disruption cascade. Elevated levels of SCFAs compromise goblet cell mucin secretion, weakening the mucosal barrier (Cordone, 2024; Strati et al., 2016). The resulting breach facilitates the translocation of pathobionts such as *Candida* and *Escherichia/Shigella*, triggering a subclinical inflammatory response marked by elevated fecal calprotectin levels and ESR (Cordone, 2024; Jiang et al., 2020; Strati et al., 2016). Dysregulated cytokine production further damages the epithelium and fosters a permissive niche for fungal proliferation, establishing a self-perpetuating loop that stabilizes the dysbiotic ecosystem (Cordone, 2024; Jiang et al., 2020; Strati et al., 2016). Thus, RTT represents a genetically anchored model disorder in which host gene dysfunction initiates a cascade of microbial, metabolic, and immunologic perturbations that become locked in *via* reciprocal feedback mechanisms, highlighting the gut as a key perpetuator of systemic pathology in monogenic neurodevelopmental disorders (Table 1 and Figure 1).

**TABLE 1:** A multi-layered comparison of gut-brain axis disruption in neurodevelopmental disorders (NDDs).

Domain of pathophysiology	Feature	ASD	ADHD	RTT
Etiological driver	Primary trigger (proposed mechanism)	“Bottom-up”: environmental/polygenic factors → bacterial dysbiosis → fungal “niche release” (Dalle et al., 2010; Dargenio et al., 2023; Strati et al., 2017; van Tilburg Bernardes et al., 2020; Yetgin, 2024; Zhang et al., 2020; Zou et al., 2021).	“Bottom-up”: initial bacterial dysbiosis, potentially linked to early-life factors → fungal expansion (Cassidy-Bushrow et al., 2023; Checa-Ros et al., 2021; Hadrich et al., 2024; Lewis et al., 2025; van Tilburg Bernardes et al., 2020; Wang L. J. et al., 2023).	“Top-down”: monogenic *MeCP2* defect → ENS dysfunction, dysmotility, and immunodeficiency → pro-dysbiotic niche (Cordone, 2024; Strati et al., 2016, 2018; Wang Q. et al., 2023).
Microbial dysbiosis	Fungal signature	Taxa: *C. albicans*↑, Saccharomycetaceae↑, Debaryomycetaceae↑ (Dargenio et al., 2023; Hughes and Ashwood, 2018; Kantarcioglu et al., 2016; Strati et al., 2017; Zou et al., 2021), Saccharomyces↓, *Aspergillus* spp. Altered (De Santis et al., 2019; Su et al., 2024) Diversity: fungal α-diversity↓ (Strati et al., 2017; Su et al., 2024).	Taxa: *Candida albicans*↑ (Hadrich et al., 2024; Lionakis et al., 2023; Wang L. J. et al., 2023). Phylum Shift: Ascomycota↑, Basidiomycota↓ (Wang L. J. et al., 2023). Diversity: variable, often showing compositional shift rather than loss of richness (Cassidy-Bushrow et al., 2023; Wang L. J. et al., 2023).	Dominant taxa: *Candida parapsilosis* (Strati et al., 2016, 2018; Yetgin, 2024). Depleted taxa: *Saccharomyces*, *Penicillium*, *Blastocystis* (non-fungal protozoan) (Hadrich et al., 2024; Strati et al., 2016; Yetgin, 2024). Diversity: Profoundly reduced fungal diversity (Strati et al., 2016; Yetgin, 2024).
	Bacterial-fungal link	Bacterial dysbiosis (e.g., altered Firmicutes/Bacteroidetes ratio) correlates with fungal changes (Dalle et al., 2010; Gaspar et al., 2025; Strati et al., 2017; Yetgin, 2024).	Bacterial disruption is hypothesized to precede and enable fungal overgrowth (Hadrich et al., 2024; Iliev and Cadwell, 2021; Leonardi et al., 2022; Lionakis et al., 2023; van Tilburg Bernardes et al., 2020; Wang L. J. et al., 2023).	Co-occurring pathobionts (*E.coli/Shigella* and *Candida*) are characteristic (Iliev and Leonardi, 2017; Jiang et al., 2020; Strati et al., 2016; Yadav et al., 2024; Zhang et al., 2022).
Gut-barrier-immune axis	Intestinal permeability	Heterogeneous; likely a feature of a patient subset with specific inflammatory profiles. Fungi can actively exacerbate it (Dalle et al., 2010; Dalton et al., 2014; de Magistris et al., 2010; Fattorusso et al., 2019; Kushak et al., 2016; Yetgin, 2024).	Plausibly increased; *in vitro* evidence demonstrates *C. albicans*-induced permeability (Hadrich et al., 2024; Iliev and Cadwell, 2021; Leonardi et al., 2022; Lionakis et al., 2023; Wang L. J. et al., 2023).	Compromised barrier function is a direct consequence of *MeCP2* defect and is exacerbated by altered SCFA profiles (Cordone, 2024; Strati et al., 2016; Yadav et al., 2024).
	Immune activation cascade	Trigger: fungal MAMPs (e.g., β-glucans) (Hughes and Ashwood, 2018; Iliev et al., 2012; McBride et al., 2023; Patin et al., 2019; Sey et al., 2024). Pathway: Dectin-1/Syk/CARD9 → Th17/IL-17 activation (Brown, 2006; Gładysz et al., 2018; Gringhuis et al., 2012; Iliev et al., 2012; Li et al., 2019; Sparber and LeibundGut-Landmann, 2019; Yao et al., 2024; Zelante et al., 2009). Systemic evidence: elevated anti-*Candida* IgG antibodies (Gładysz et al., 2018; Hughes and Ashwood, 2018).	Trigger: fungal MAMPs (Lionakis et al., 2023; McBride et al., 2023; Patin et al., 2019; Sey et al., 2024). Pathway: CLR/Syk/CARD9 → Th17/IL-17 activation (Hadrich et al., 2024; Leonardi et al., 2022; Lionakis et al., 2023). Systemic evidence: correlates with inflammatory markers (Checa-Ros et al., 2021; Hadrich et al., 2024; Lewis et al., 2025; Wang L. J. et al., 2023).	Trigger: pathobiont translocation through compromised barrier (Cordone, 2024; Strati et al., 2016; Strati et al., 2018). Pathway: *MeCP2*-dependent dysregulation of Th1/Th17 response; aberrant IL-10 production (Cordone, 2024; Strati et al., 2016, 2018). Systemic evidence: elevated fecal calprotectin, ESR, serum IgA (Cordone, 2024; Strati et al., 2016, 2018).
Metabolic and neuroactive disruption	SCFA profile	Butyrate↓, PPA↑ (Liu et al., 2019; Wang et al., 2012).	Altered SCFA profiles secondary to interkingdom dysbiosis (Checa-Ros et al., 2021; Hadrich et al., 2024; Lewis et al., 2025; van Tilburg Bernardes et al., 2020; Wang L. J. et al., 2023).	Markedly propionate, isobutyrate↑, and isovalerate↑ (Borghi and Vignoli, 2019; Cordone, 2024; MacFabe, 2015; Nankova et al., 2014; Strati et al., 2016).
	Fungal-derived metabolites	Ammonia: elevated fecal levels, linked to neurotoxicity (Wang et al., 2012). Other toxins: potential contribution from D-arabinose isomers (Hadrich et al., 2024).	Indirectly influences CNS via cytokine-mediated modulation of neurotransmitter synthesis (e.g., dopamine, serotonin) (Borrajo et al., 2014; Channer et al., 2023; Checa-Ros et al., 2021; Felger and Lotrich, 2013; Goldsmith et al., 2023; Lee et al., 2018; Lewis et al., 2025; Lewitus et al., 2014; Miller et al., 2017).	Contributes to the neurotoxic milieu alongside bacterially-derived PPA and ammonia within the “multi-hit” framework (Cordone, 2024; MacFabe, 2015; Nankova et al., 2014; Strati et al., 2016).
Translational implications	Therapeutic target	Multi-kingdom ecosystem modulation. Management of neurotoxic metabolites.	Targeting early-life bacterial-fungal interactions. Mitigation of systemic inflammation (Checa-Ros et al., 2021; Hadrich et al., 2024; Lewis et al., 2025; Wang L. J. et al., 2023).	Primary: restoration of gut motility. Secondary: addressing downstream dysbiosis (Cordone, 2024; Strati et al., 2016).

## 6 Discussion

**FIGURE 2:**
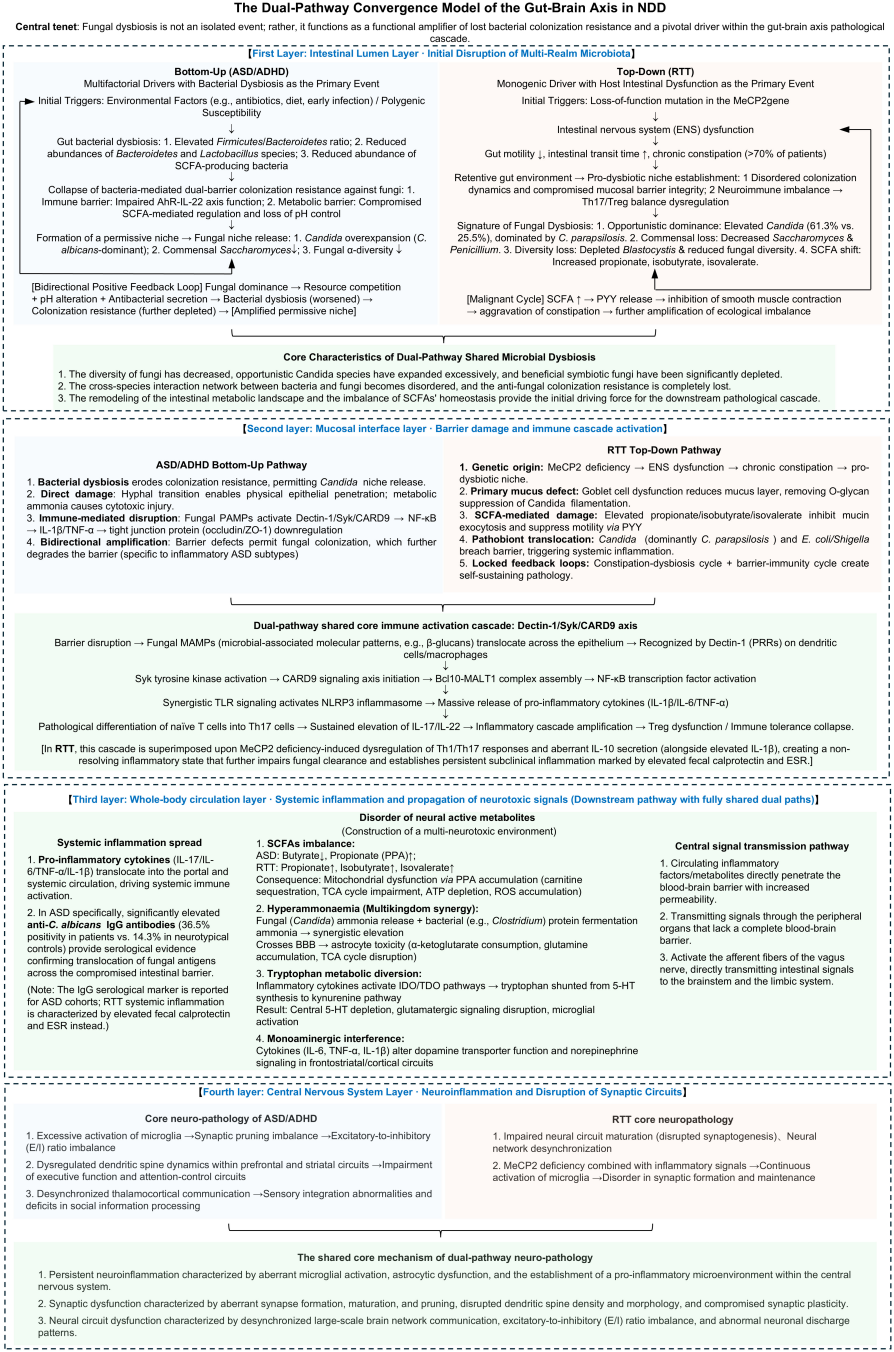
Schematic of the bacteria-fungi-host tripartite interaction framework and the dual-pathway convergent model underlying NDDs. This schematic illustrates a vertically integrated, multi-layered mechanistic cascade driving the pathogenesis of neurodevelopmental disorders (NDDs) via the microbiota-gut-brain axis, highlighting two etiologically distinct but downstream-convergent pathways: (1) Bottom-up pathway (predominant in ASD and ADHD): Initiated by multifactorial environmental and polygenic susceptibility, leading to primary bacterial dysbiosis, loss of fungal colonization resistance, and subsequent fungal niche release. (2) Top-down pathway (characteristic of RTT): Driven by monogenic MeCP2 loss-of-function mutations, triggering enteric nervous system dysfunction, chronic constipation, and a pro-dysbiotic luminal niche. Both pathways converge on a shared core pathological cascade: fungal dysbiosis (dominated by opportunistic Candida overgrowth and depleted beneficial fungi) → intestinal mucosal barrier disruption → activation of the conserved Dectin-1/Syk/CARD9/Th17 immune axis → systemic inflammation and neuroactive metabolite imbalance → propagation of neuroinflammatory and cytotoxic signals to the central nervous system → synaptic dysfunction, neural circuit disruption, and core neuropathological features of NDDs. The mechanisms and data presented in this schematic are supported by evidence cited in the main text.

In synthesizing the evidence, this review has focused on two of the most prevalent and heterogeneous NDDs—ASD and ADHD—as primary models for the “bottom-up,” multifactorial dysbiosis pathway. RTT is examined in parallel as a clarifying contrast, providing a defined “top-down” model of genetic origin. Importantly, the mechanisms discussed—spanning intestinal barrier disruption, immune activation, and neuroactive metabolite imbalance—do not merely explain discrete diagnostic labels. They converge on fundamental processes of brain development and function, including synaptogenesis, myelination, and neural circuit refinement (Alkuwaiti et al., 2025; Louveau et al., 2023). Consequently, the “bacteria-fungi-host” interaction framework advanced here holds significant implications for understanding ID, a core and often shared functional impairment across many NDDs (Biagi et al., 2014; Han et al., 2025; Protic et al., 2025). Notably, ID represents the common endpoint of disrupted neurodevelopmental trajectories, where gut-derived metabolites such as SCFAs and neurosteroids may modulate cognitive severity through epigenetic regulation of synaptic plasticity and mitochondrial energy metabolism (Han et al., 2025; Protic et al., 2025; Figures 1, 2).

The existing body of research into gut dysbiosis in NDDs has largely focused on bacterial or fungal imbalances in isolation, yet the evidence synthesized in this review points to the indispensable role of their interplay (Dalle et al., 2010; Strati et al., 2016; Yetgin, 2024; Zhang et al., 2020). This review therefore aims to consolidate “multikingdom interactions” as a central explanatory paradigm for understanding the pathogenesis of gut ecosystem disruptions in individuals with NDDs.

This framework is best conceptualized as a tripartite “bacteria–fungi–host” interaction, where the components are interconnected in a feedback loop rather than a simple linear sequence. Within this model, fungal dysbiosis, such as the overgrowth of *Candida*, should not be viewed as an independent event but as a functional “amplifier” and “effector” of an underlying bacterial imbalance. The initial loss of bacterial colonization resistance—driven by a depletion of key commensal taxa—creates a permissive niche that fungi exploit, thereby amplifying the primary bacterial dysbiosis and its pathophysiological consequences. For instance, in both patients with RTT and ASD, primary bacterial dysbiosis leads to reduced production of SCFAs and subsequent immune dysregulation (Liu et al., 2019; Strati et al., 2016; Xiang et al., 2025). These changes create an environment conducive to fungal overgrowth, which in turn exacerbates the initial condition by reinforcing gut barrier dysfunction and promoting proinflammatory responses, such as increased Th17 activation (Iliev et al., 2012; Sparber and LeibundGut-Landmann, 2019; Zelante et al., 2009). This integrated model provides a more robust and mechanistically coherent foundation for interpreting the complex gut-brain axis disruptions observed in individuals with NDDs.

A consistent signature of gut mycobiota dysbiosis is evident across the neurodevelopmental disorders of ASD, ADHD, and RTT. This shared pattern is primarily characterized by reduced fungal diversity and a significant overrepresentation of opportunistic *Candida* species, particularly *C. albicans* and, in patients with RTT, *C. parapsilosis* (Strati et al., 2016, 2017; Wang L. J. et al., 2023). While the foregoing discussion has emphasized the enrichment of Candida species across ASD, ADHD, and RTT, it is critical to recognize that gut mycobiota alterations in NDDs are characterized by bidirectional changes: the expansion of opportunistic fungi coincides with the depletion of potentially beneficial taxa. For instance, *Saccharomyces cerevisiae* —a fungus with established immunomodulatory properties—is significantly reduced in patients with RTT and severe ASD (Borghi and Vignoli, 2019; De Santis et al., 2019; Su et al., 2024). Similarly, *Aspergillus* species, which are detected in neurotypical controls, show altered abundance patterns in ASD (De Santis et al., 2019; Su et al., 2024). Moreover, recent experimental evidence demonstrates that Candida albicans itself can establish mutualistic relationships with the host, regulating microbial dynamics and metabolic homeostasis without causing pathology (Peroumal et al., 2022). These observations challenge the notion of fungi as purely adversarial agents and support a model in which dysbiosis represents both the loss of beneficial fungi and the gain of potentially harmful ones (Wu et al., 2025). Future therapeutic strategies should therefore consider not only the suppression of pathogenic fungal overgrowth but also the restoration of commensal fungal communities that reinforce intestinal barrier function and immune tolerance. This fungal imbalance is mechanistically linked to a common set of downstream pathological consequences, including compromised intestinal barrier integrity (“leaky gut”), the establishment of a subclinical, systemic inflammatory state driven by the Th17/IL-17 pathway, and disruptions in the production of key microbial metabolites like short-chain fatty acids (SCFAs) (Dalle et al., 2010; Hadrich et al., 2024; Yetgin, 2024; Zhang et al., 2020). It is important to note, however, that our understanding of this dysregulated metabolic landscape is uneven across disorders. While alterations in SCFAs and certain neurotransmitters have been documented in ASD, the specific roles and alterations of other crucial neuroactive metabolites—such as GABA, biotin, and dopamine precursors—in ADHD and RTT remain largely uncharted (Giuliano and Tognini, 2025; Wang X. et al., 2025). The translocation of fungal components such as β-glucans appears to be a shared trigger, initiating a conserved immune cascade via the Dectin-1/Syk/CARD9 signaling axis that perpetuates this low-grade inflammation (Iliev et al., 2012; Lionakis et al., 2023). Despite these shared features, the etiological pathways driving this dysbiosis appear to diverge, primarily due to the distinct origins of the disorders. In patients with RTT, the evidence points to a “top-down” cascade initiated by a single-gene defect. Loss-of-function mutations in the *MeCP2* gene directly impair the ENS, leading to severe gut dysmotility and chronic constipation (Cordone, 2024; Strati et al., 2016). This genetically determined gastrointestinal environment creates a stable, pro-dysbiotic niche that secondarily promotes bacterial and fungal imbalances. In contrast, for the multifactorial disorders ASD and ADHD, the evidence suggests a corresponding “bottom-up” process. In patients with these conditions, a combination of environmental factors and polygenic susceptibility is thought to first drive bacterial dysbiosis, which then erodes the colonization resistance that normally constrains fungal populations (Strati et al., 2017; van Tilburg Bernardes et al., 2020; Wang L. J. et al., 2023). This change leads to “niche release,” allowing for the opportunistic expansion of fungi like *Candida*. These distinct initiating pathways—a primary, genetically driven gut dysfunction in individuals with RTT versus secondary fungal overgrowth following bacterial dysbiosis in individuals with ASD/ADHD—have significant translational implications, suggesting that therapeutic strategies may need to be tailored accordingly. This could involve managing primary gut motility in RTT, versus interventions designed to correct the “bottom-up” dysbiosis characteristic of ASD and ADHD, such as targeting the broader bacterial–fungal ecosystem.

Therefore, despite their distinct etiological origins, both the “top-down” and “bottom-up” pathways converge on a set of shared gut-brain axis disruptions. The chronic neuroinflammation and synaptic dysregulation arising from these pathways are established contributors to cognitive impairment across NDDs (Alkuwaiti et al., 2025; Louveau et al., 2023). Specifically, elevated pro-inflammatory cytokines (e.g., IL-6, TNF-α) and microglial hyperactivation disrupt neural circuit maturation, while gut mycobiota-derived metabolites—including neurosteroids such as dehydroepiandrosterone sulfate and microbially-modulated tryptophan metabolites—regulate neurotransmitter balance and neuroplasticity (Han et al., 2025; Protic et al., 2025). These converging mechanisms position gut mycobiota stability as a potential modulator of ID risk across diverse NDDs, offering a transdiagnostic target for intervention (Table 1).

The compelling associative data synthesized in this review strongly implicate gut mycobiota dysbiosis in the pathophysiology of NDDs; however, we emphasize that these findings represent associations rather than established causal relationships. However, a rigorous critique of the current evidence base is essential to define the limitations of the field and guide future research. The most significant constraint is the fundamentally correlative nature of the existing human studies, which cannot definitively establish the directionality of the relationship between fungal dysbiosis and NDDs. Based on the mechanistic evidence synthesized in this review, we propose a hypothetical bidirectional model in which: (i) primary fungal dysbiosis could potentially act as a disease-modifying factor by driving systemic immune activation and metabolic disruptions that may exacerbate neurodevelopmental impairments—this is supported by animal models where fungal colonization or metabolite administration induces ASD-like behaviors, though translation to human causality requires further validation (Hughes and Ashwood, 2018; Thomas et al., 2010); and (ii) NDD-related physiological alterations (e.g., gut dysmotility in RTT, dietary restrictions, medication use) can secondarily promote fungal overgrowth, creating a self-perpetuating cycle (Strati et al., 2016; Zou et al., 2021). This reciprocal causation framework is exemplified by the contrasting etiological pathways observed across disorders: in RTT, a genetically determined “top-down” cascade from MeCP2 deficiency initiates gut dysmotility, which precedes and drives dysbiosis (Strati et al., 2016; Wang Q. et al., 2023); conversely, in ASD and ADHD, environmental and polygenic factors likely trigger “bottom-up” bacterial dysbiosis first, which then erodes colonization resistance and permits fungal niche release (Dargenio et al., 2023; Hughes and Ashwood, 2018; Wang L. J. et al., 2023). A critical, yet under-explored, component of this bidirectional model is the specific suite of microbial metabolites that ultimately mediate the brain effects. The “bottom-up” pathway in ASD and ADHD, and the disrupted microbial ecology in RTT, are both predicted to distort the production of a wider array of neuroactive compounds beyond SCFAs, including GABA, biotin, and dopamine precursors. Currently, direct evidence linking fungal dysbiosis to these specific metabolic disturbances is strongest in ASD models and severely lacking for ADHD and RTT. Thus, rather than viewing fungal dysbiosis as exclusively a cause or consequence, we posit that it functions as both an active contributor to and a downstream marker of NDD pathophysiology, with the predominant directionality varying by disorder subtype and individual patient factors. Future research must move beyond correlations and employ strategies that can infer causality. The monogenic nature of RTT, where a “top-down” cascade from a known genetic defect precedes dysbiosis, provides a unique model to investigate these temporal dynamics (Strati et al., 2016). This analysis could be complemented by prospective birth cohort studies that track mycobiota development from infancy relative to a later NDD diagnosis, and interventional experiments in animal models, such as fecal microbiota transplantation from human donors or specific fungal colonization, to directly test causal effects on behavior and neurobiology. A further challenge lies in the heterogeneity and ongoing controversy surrounding the “leaky gut” hypothesis. The present review summarizes conflicting evidence, as detailed in section “3.2.1 Intestinal permeability and barrier function: heterogeneity and fungal involvement”; while early reports indicated a significantly higher prevalence of abnormal intestinal permeability in children with ASD (de Magistris et al., 2010; Fattorusso et al., 2019), subsequent well-controlled investigations found no significant differences (Dalton et al., 2014; Kushak et al., 2016). This inconsistency strongly suggests that a state of measurably compromised intestinal barrier function is not a ubiquitous feature of ASD (or likely other NDDs) but rather a distinct pathological trait pertinent to a subset of individuals. This observation reframes the “leaky gut” hypothesis from a disorder-wide theory to a framework for patient stratification. Fungus-driven barrier disruption may be particularly relevant in individuals with specific microbial, inflammatory, or clinical profiles. Therefore, future research should move beyond broad case-control comparisons of permeability and instead focus on identifying and validating biomarkers (e.g., specific fungal-bacterial consortia, host immune signatures) that define this vulnerable subgroup, for whom barrier-centric interventions might be most meaningful. Beyond the correlational design limitations discussed above, the field faces substantial technical heterogeneity that obscures causal pathways and impedes reproducibility. Unlike bacterial 16S sequencing, which has achieved relative standardization through the Earth Microbiome Project protocols, fungal ITS sequencing lacks consensus on target regions (ITS1 versus ITS2) and primer selection. This variability introduces systematic technical biases: for example, ITS1 primers preferentially amplify Ascomycota (including *Candida* and *Aspergillus*) while potentially under-representing Basidiomycota (e.g., *Malassezia*). Consequently, the observed enrichment of *Candida* in ASD (Strati et al., 2017; Zou et al., 2021) versus its variable detection in ADHD (Wang L. J. et al., 2023) may partially reflect primer bias rather than disorder-specific biology. Similarly, the reduced fungal α-diversity reported in RTT (Strati et al., 2016) requires cautious interpretation, as low-biomass samples are prone to contamination artifacts that mimic diversity loss. Moreover, the ITS region lacks the standardization of the 16S rRNA gene, and fungal annotation relies on the comparatively limited UNITE database, which can hinder accurate taxonomic classification and functional inference (Sam et al., 2017; Strati et al., 2017). Critically, cross-disorder comparisons presented in this review—such as the proposed shared *Candida* signature across NDDs (Table 2) —must be viewed as preliminary patterns awaiting validation through methodologically harmonized studies employing identical ITS regions, inclusion of mock communities, negative blanks, and quantitative spike-in controls. Future longitudinal studies must prioritize such standardization to disentangle true biological signals from technical noise.

**TABLE 2:** A systematic overview of gut mycobiota in neurodevelopmental disorders: species, associations, and pathogenic implications.

Fungal species	Classification	Associated NDDs	Proposed interaction with bacteria	Proposed interaction with host / pathogenic mechanism	Key references
*Candida albicans*	Opportunistic pathobiont (Ascomycota)	ASD, ADHD, RTT	Competitor: exploits niche released by bacterial dysbiosis (e.g., loss of *Lactobacillus*, *Bifidobacterium*). Bacterial SCFA depletion reduces inhibition of its growth. (*) In Zou et al. (2021), abundance was 2.24% in ASD vs. 3.93% in controls (not significantly different); however, Strati et al. (2017) reported significantly elevated *Candida* genus abundance in ASD (37.7% vs. 14.1%, *P* = 0.09).	Immune activator and barrier disruptor: β-glucans/MAMPs trigger Dectin-1/Syk/CARD9 pathway → Th17/IL-17 activation→ systemic inflammation. Hyphal transition damages epithelium and compromises tight junctions. May produce ammonia; however, direct evidence linking fungal-derived ammonia to neurotoxicity in ASD specifically requires further investigation.	Acosta-Rodriguez et al., 2007; Brown, 2006; Bush, 2010; Dalle et al., 2010; Dargenio et al., 2023; Deveau et al., 2018; Ene et al., 2014; Gaspar et al., 2025; Gładysz et al., 2018; Gringhuis et al., 2012; Hadrich et al., 2024; Hughes and Ashwood, 2018; Iliev and Leonardi, 2017; Iliev et al., 2012; Kantarcioglu et al., 2016; Lionakis et al., 2023; McBride et al., 2023; McCrory et al., 2023; Parada Venegas et al., 2019; Patin et al., 2019; Ríos-Covián et al., 2016; Sey et al., 2024; Strati et al., 2016; Strati et al., 2017, 2018; Wang et al., 2012; Wang L. J. et al., 2023; Yetgin, 2024; Zou et al., 2021
*Candida parapsilosis*	Opportunistic pathobiont (Ascomycota)	RTT (dominant)	Co-pathogen: thrives in the costive, dysbiotic gut environment characteristic of RTT, often co-occurring with proteobacterial pathobionts (E. coli/Shigella*).	Virulence and immune dysregulator: exhibits enhanced biofilm formation in RTT. Triggers an aberrant cytokine profile (elevated IL-1β and IL-10), contributing to persistent sub-inflammatory state. The clinical significance of antifungal resistance in this context remains to be fully elucidated.	Cordone, 2024; Iliev and Leonardi, 2017; Jiang et al., 2020; Strati et al., 2016, 2018; Yadav et al., 2024; Yetgin, 2024; Zhang et al., 2022
*Candida krusei*	Opportunistic pathogen (Ascomycota)	ASD	Opportunistic colonizer: (*) identified in ASD samples (20% of isolates); not detected in healthy controls. May expand in the context of bacterial dysbiosis, potentially contributing to increased fungal burden.	Pathogen: intrinsically fluconazole-resistant. *May* synergize with other Candida* species in driving dysbiosis; this hypothesis requires experimental validation.	Kantarcioglu et al., 2016
*Candida glabrata*	Opportunistic pathogen (Ascomycota)	ASD	Opportunistic colonizer: identified in ASD samples (15% of isolates); not detected in healthy controls. Co-occurs with other Candida* species in dysbiotic gut; may expand when bacterial colonization resistance is compromised.	Pathogen: (*) shows elevated MICs to fluconazole. May contribute to cumulative fungal burden; its specific pathogenic role in ASD remains to be determined.	Kantarcioglu et al., 2016
*Saccharomyces cerevisiae*	Opportunistic yeast (Ascomycota)	ASD↑	Opportunistic colonizer: significantly increased in ASD (58.38% vs. 36.72% in controls, *P* = 0.032). Detected in ASD samples but not in healthy controls in some studies.	Immunomodulator: may enhance TNF-α and IL-6 production upon stimulation. Its role in ASD pathogenesis is complex; while considered potentially harmful in some contexts, its variant *S. boulardii* is used as a probiotic for gastrointestinal complications in autism.	Kantarcioglu et al., 2016; Zou et al., 2021
***Saccharomyces*** spp. (other)	Opportunistic yeast (Ascomycota)	ASD↑	Symbiont: (*) significantly increased in ASD at genus level (58.41% vs. 36.84%, *P* = 0.032).	Ecological indicator: higher abundance in ASD; may reflect altered gut ecosystem. Its specific role (beneficial vs. harmful) in ASD context requires further investigation.	Zou et al., 2021
*Aspergillus versicolor*	Environmental/commensal (Ascomycota)	ASD↓	Community member: (*) almost absent in ASD (0.01%) compared to controls (1.78%).	Metabolic modulator: produces metabolites with anti-inflammatory activities; its depletion may reflect immune dysbiosis in ASD.	De Santis et al., 2019; Su et al., 2024; Zou et al., 2021
*Aspergillus* spp. (other)	Environmental/commensal (Ascomycota)	ASD↓	Community member: significantly reduced in ASD at genus level (0.66% vs. 3.52%, *P* = 0.0058). Multiple species (*A. flavus*, *A. nidulans*, *A. luchuensis*) altered in ASD.	Metabolic contributor: some species produce neuroactive metabolites; may contribute to metabolic dysregulation in the context of fungal dysbiosis.	De Santis et al., 2019; Su et al., 2024; Zou et al., 2021
*Penicillium* spp.	Beneficial commensal (Ascomycota)	RTT↓	Symbiont: (*) co-exists with beneficial bacterial taxa; depleted alongside bacterial commensals in RTT.	Indicator of dysbiosis: (*) depletion reflects profound ecosystem degradation and loss of fungal diversity in RTT.	Strati et al., 2016; Yetgin, 2024
*Blastocystis* spp.	Protozoan-like eukaryote	RTT↓	Symbiont: (*) part of normal gut microbiome; co-exists with diverse bacterial and fungal communities.	Indicator of severe dysbiosis: (*) near-complete absence in RTT indicates profoundly disrupted gut ecosystem.	Strati et al., 2016
*Rhodotorula* spp.	Environmental yeast (Basidiomycota)	ADHD (early-life marker)	Early colonizer: (*) part of initial gut fungal colonization; interacts with developing bacterial community.	Developmental influencer: (*) early-life fungal signature that correlates with later ADHD risk. May influence immune and neurodevelopmental trajectories; causal relationships remain to be established.	Cassidy-Bushrow et al., 2023
*Malassezia* spp.	Skin-associated yeast (Basidiomycota)	ADHD (early-life marker)	Early colonizer: (*) early-life gut presence may reflect translocation or initial colonization patterns; interacts with bacterial community.	Developmental predictor: (*) early-life fungal signatures correlate with later ADHD diagnosis. May serve as biomarker for neurodevelopmental risk; predictive utility requires prospective validation.	Cassidy-Bushrow et al., 2023

This table summarizes key fungal species implicated in neurodevelopmental disorders (NDDs) and their proposed roles within the bacteria-fungi-host interaction framework. Symbol definitions: Upward arrow indicates that the fungal taxon is enriched or shows increased abundance in the specified NDD compared to neurotypical controls. Downward arrow indicates that the fungal taxon is depleted or shows decreased abundance in the specified NDD compared to neurotypical controls.

(*) denotes established findings that are directly supported by experimental or clinical evidence from the cited references. Statements without (*) represent proposed mechanisms, hypotheses, or associations derived from indirect evidence, extrapolation from related fields, or theoretical frameworks that require further experimental validation. ^†^While this table focuses primarily on fungal taxa, *Blastocystis* (listed under Classification as ‘Protozoan-like eukaryote’) is included due to its co-detection in metataxonomic sequencing and its ecological relevance in RTT gut dysbiosis.

Before transitioning to therapeutic perspectives, it is crucial to frame the existing evidence within a hierarchical and causal context. While compelling associative data link specific gut mycobiota signatures (e.g., reduced diversity, *Candida* overgrowth Table 2) to NDDs and related immunometabolic disturbances (Hadrich et al., 2024; Hughes and Ashwood, 2018; Strati et al., 2016, 2017; Wang L. J. et al., 2023; Yetgin, 2024), and mechanistic studies in preclinical models detail plausible pathways involving barrier dysfunction, Dectin-1/Th17 activation, and metabolite imbalance (Dalle et al., 2010; Iliev et al., 2012; Lionakis et al., 2023; Zelante et al., 2009), direct evidence for causality in humans and for the efficacy of fungus-targeted interventions remains in its infancy. Currently, clinical intervention studies in NDD populations are scarce, typically small-scale, and rarely designed to isolate the effects of modulating the fungal community or multikingdom interactions (Aslin et al., 2023; Checa-Ros et al., 2021; Chui et al., 2024; Hadrich et al., 2024; Takyi et al., 2025; Wang Q. et al., 2023). Consequently, the therapeutic strategies discussed hereafter must be interpreted as a hypothesis-generating framework and a roadmap for future validation, rather than as established clinical guidelines. The translation of this mechanistic rationale into practice is contingent upon rigorously bridging this significant evidence gap through targeted biomarker stratification and controlled interventional trials.

The mechanistic pathways elucidated in this review, particularly those involving multikingdom interactions and the gut-barrier-immune axis, provide a translational roadmap for advancing NDD management. A priority along this roadmap must be to move beyond associative signatures and define the functional metabolic output of dysbiotic ecosystems. This necessitates closing the striking knowledge gap regarding key neuroactive metabolites—GABA, biotin, dopamine precursors—in ADHD and RTT. Future studies should specifically test whether the fungal dysbiosis observed in these disorders drives, or is associated with, quantifiable alterations in the bacterial production and host circulation of these compounds. Such work would not only illuminate disorder-specific pathophysiology but also directly test the central tenet of the “bacteria-fungi-host” framework: that fungal perturbations exert their neurodevelopmental impact in large part by reshaping the metabolic landscape defined by bacteria. A critical next step is the validation of mycobiota-based biomarkers, such as fungal abundance ratios (e.g., *Candida* to *Saccharomyces* ratio), fecal metabolites like ammonia, and host immune responses including anti-*Candida* IgG, to identify patient subsets with distinct gut ecosystem profiles. If validated in prospective cohorts, such biomarkers would prospectively guide precision therapeutic strategies. For instance, identifying a “*Candida*-enriched” subtype could facilitate the selection of patients for future clinical trials evaluating targeted antifungal therapy, assessing its efficacy and safety against placebo or standard care. Similarly, microbial and metabolic profiles could be explored to design dietary interventions aimed at modulating nutrient availability and SCFA balance, or to develop multikingdom modulators like specific polyphenols. It must be emphasized that the efficacy and safety of all such strategies would ultimately require confirmation in rigorously designed randomized controlled trials. Key mechanistic questions, including whether gut fungi influence neurodevelopment via epigenetic mechanisms or produce unique neuroactive metabolites, must be resolved through longitudinal studies and gnotobiotic models to fully realize this potential. In summary, the integration of biomarker discovery, the exploration of targeted interventions, and ongoing mechanistic clarification holds the promise of progressively advancing the field toward a future in which modulation of the gut mycobiota could contribute to personalized NDD therapeutics. The essential first step toward this goal is to rigorously address the current formidable gap between associative evidence and demonstrated clinical efficacy.
